# RING Zinc Finger Proteins in Plant Abiotic Stress Tolerance

**DOI:** 10.3389/fpls.2022.877011

**Published:** 2022-04-14

**Authors:** Guoliang Han, Ziqi Qiao, Yuxia Li, Zongran Yang, Chengfeng Wang, Yuanyuan Zhang, Lili Liu, Baoshan Wang

**Affiliations:** ^1^Shandong Provincial Key Laboratory of Plant Stress Research, College of Life Science, Shandong Normal University, Jinan, China; ^2^Dongying Institute, Shandong Normal University, Dongying, China

**Keywords:** RING zinc finger proteins, plant, abiotic stress, E3 ubiquitin ligase, regulation mechanism

## Abstract

RING zinc finger proteins have a conserved RING domain, mainly function as E3 ubiquitin ligases, and play important roles in plant growth, development, and the responses to abiotic stresses such as drought, salt, temperature, reactive oxygen species, and harmful metals. RING zinc finger proteins act in abiotic stress responses mainly by modifying and degrading stress-related proteins. Here, we review the latest progress in research on RING zinc finger proteins, including their structural characteristics, classification, subcellular localization, and physiological functions, with an emphasis on abiotic stress tolerance. Under abiotic stress, RING zinc finger proteins on the plasma membrane may function as sensors or abscisic acid (ABA) receptors in abiotic stress signaling. Some RING zinc finger proteins accumulate in the nucleus may act like transcription factors to regulate the expression of downstream abiotic stress marker genes through direct or indirect ways. Most RING zinc finger proteins usually accumulate in the cytoplasm or nucleus and act as E3 ubiquitin ligases in the abiotic stress response through ABA, mitogen-activated protein kinase (MAPK), and ethylene signaling pathways. We also highlight areas where further research on RING zinc finger proteins in plants is needed.

## Introduction

Plants face a variety of abiotic stresses, such as drought, high salinity, cold, and heat stress, during their growth and development (Li et al., 2019). Under these adverse conditions, plants suffer various degrees of damage. For example, under high-salt stress, the imbalance of Na^+^/K^+^ inside and outside the cell restricts plant growth (Assaha et al., 2017) and the stress-induced accumulation of ROS causes oxidative stress (Apel and Hirt, 2004). Plants have evolved a variety of physiological and biochemical defense mechanisms to deal with abiotic stressors (Hasanuzzaman et al., 2013), such as antioxidant defenses (Yang and Guo, 2018), programmed cell death (Locato and De Gara, 2018), stomatal movement (Qiao et al., 2016), and autophagy (Su et al., 2020). Functional proteins and transcription factors such as MYBs (Li C. et al., 2015), bHLHs (Sun et al., 2018), WRKYs (Chen et al., 2012), and zinc finger proteins (Han et al., 2020b,^2021^) are involved in these defense mechanisms.

Zinc finger proteins maintain a finger-like spatial configuration by binding to zinc ions through amino acids in the peptide chain, and are widely distributed in eukaryotes (Han et al., 2020a). RING zinc finger proteins have been identified in many plants, including *Arabidopsis thaliana* (Linden et al., 2021; Liu et al., 2021; Van Nguyen et al., 2021), rice (*Oryza sativa*) (Kim et al., 2020; Kim and Jang, 2021; Zhu et al., 2021), maize (*Zea mays*) (Gao et al., 2012; Xia et al., 2012; Yang et al., 2018), pepper (*Capsicum annuum*) (Liu et al., 2007; Joo et al., 2019a,^2020^), wheat (*Triticum aestivum*) (Agarwal and Khurana, 2018; Liu et al., 2018; Wang et al., 2020), potato (*Solanum tuberosum*) (Guo et al., 2003; Ni et al., 2010; Qi et al., 2020), wild tomato (*Solanum pimpinellifolium*) (Yang et al., 2016), wild Chinese grape (*Vitis pseudoreticulata*) (Yu et al., 2013; Wang et al., 2017), soybean (*Glycine max*) (Du et al., 2010; Biniek et al., 2017; Zhao et al., 2020), *Brassica rapa* (Jung et al., 2013), tobacco (*Nicotiana benthamiana)* (Wu et al., 2014), and cassava (*Manihot esculenta* Crantz) (Dos Reis et al., 2012). RING zinc finger proteins have a characteristic RING zinc finger domain. They bind to specific gene sequences, interact with a variety of proteins, participate in signal transduction and gene expression, and mediate growth, development, and environmental adaptation (An et al., 2017c; Sun et al., 2019; Han et al., 2020b). RING zinc finger proteins regulate photomorphogenesis (Von Arnim and Deng, 1993; Hardtke et al., 2002; Yang et al., 2015), floral organ size (Disch et al., 2006), rosette leaf shape (Wang et al., 2018), root development (Karlowski and Hirsch, 2003; Fleury et al., 2007; Liu et al., 2007), flowering time (Lee et al., 2001; Chen and Ni, 2006), fruit development (Wu et al., 2014), and symbiotic nodulation (Cai et al., 2018). In addition, RING zinc finger proteins play an important role in abiotic stress tolerance (Lu, 2017), especially in drought, temperature, salt, and ROS stresses (Kim et al., 2019).

Here, we focus on the role of RING zinc finger proteins in plant responses to abiotic stress and provide suggestions for future research.

## Structure and Domain Organization of Ring Zinc Finger Proteins

The RING finger domain of RING zinc finger proteins is the site of protein–protein interaction and is necessary for its E3 ligase activity. RING zinc finger proteins contain a conserved cysteine-rich ring finger domain (RING finger domain), which consists of 40–60 residues arranged thus: Cys-X_2_-Cys-X_9–39_-Cys-X_1–3_-His-X_2–3_-Cys/His-X_2_-Cys-X_4–48_-Cys-X_2_-Cys (Stone et al., 2005). The RING finger domain contains eight spatially conserved Cys and His residues that act as metal ligands to chelate two zinc ions, and form a staggered secondary structure as a platform for the ubiquitin conjugating enzyme E2.

## Classification of Ring Zinc Finger Proteins

RING zinc finger proteins are mainly divided into the following 13 types: RING-H2, RING-HC (RING-HCa and RING-HCb), RING-v, RING-C2, RING-D, RING-S/T, RING-G, RING-mh2, RING-mhc, C2SHC4, C3GC3S, C2HC5, and C3HCHC2 (Sun et al., 2019). RING zinc finger proteins have been isolated and identified in many plants. For instance, 508 RING zinc finger domains have been predicted in *Arabidopsis thaliana* and classified into seven categories (Jiménez-López et al., 2018). In *Brassica rapa*, 731 RING domains were identified from 715 predicted RING zinc finger protein genes and classified into eight categories (Alam et al., 2017). In tomato (*Solanum lycopersicum*), 474 RING domains were identified from 469 predicted RING zinc finger proteins and classified into seven categories (Yang et al., 2019). In rice, 425 RING zinc finger protein genes were identified and classified into four categories (Lim et al., 2010). In apple (*Malus domestica*), 688 RING domains were identified and divided into nine types (Li Y. et al., 2011). In *Brassica oleracea*, 756 RING domains were identified from 734 predicted RING zinc finger proteins and divided into eight types (Yang and Lu, 2018). In *Ostreococcus tauri*, the smallest photosynthetic eukaryote yet discovered, only 65 RING finger domains were identified from 65 predicted RING zinc finger proteins and divided into eight RING domain types (Gao et al., 2016). The specific structure domain characteristics and number were showed in Supplementary Table 1. According to the above reports, RING-HC (C3HC4) and RING-H2 (C3H2C3) types are the most abundant. The main difference between RING-HC and RING-H2 types is that the fifth Cys/His residue in the common sequence is Cys or His, respectively (Stone et al., 2005). It can be speculated that RING-HC and RING-H2 types of RING zinc finger proteins play important roles in plant growth and development and adversity stress.

## Subcellular Localization of Ring Zinc Finger Proteins

**FIGURE 1:**
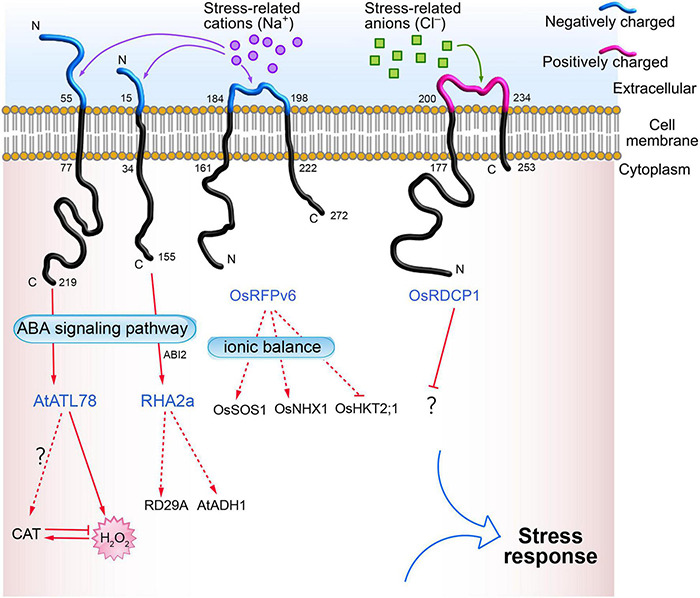
Bioinformatics analysis of transmembrane domains of RING zinc finger proteins located on the plasma membrane. The online tools TMHMM Server v. 2.0 (http://www.cbs.dtu.dk/services/TMHMM/) and Expasy ProtParam (https://web.expasy.org/protparam/) were used to for the analysis.

Plant RING zinc finger proteins are located in the plasma membrane, cytoplasm, and nucleus. RING zinc finger proteins located in the plasma membrane with a characteristic transmembrane domain include ABA INSENSITIVE RING PROTEIN 1 (AtAIRP1) (Ryu et al., 2010), RING-H2 FINGER A2A (RHA2a) (Li H. et al., 2011), and ARABIDOPSIS TÓXICOS EN LEVADURA 78 (AtATL78) (Kim S. J. and Kim W. T., 2013) in Arabidopsis; RING DOMAIN-CONTAINING PROTEINS 1 (OsRDCP1) (Bae et al., 2011), SALT-INSENSITIVE RING H2-TYPE 14 (OsSIRH2-14) (Park et al., 2019), and RING FINGER PROTEIN V6 (OsRFPv6) (Kim et al., 2021) in rice; VpRH2 in grape (Wang et al., 2017); LjCZF1 in *Lotus japonicus* (Cai et al., 2018); and StRFP2 in potato (Qi et al., 2020). Some of the transmembrane RING zinc finger proteins (such as AtATL78, RHA2a, OsRFPv6, and OsRDCP1) involved in abiotic stress are shown in Figure 1.

RING zinc finger proteins located in the cytoplasm include AtAIRP4 (Yang et al., 2016) and ERAD-mediating RING finger protein (EMR) (Park et al., 2018a) in Arabidopsis; OsSIRH2-14 and OsSIRP1 (Hwang et al., 2016) in rice; ABA SENSITIVE RING FINGER E3 LIGASE 1 (CaASRF1) (Joo et al., 2019a) and DROUGHT SENSITIVE RING FINGER PROTEIN 1 (CaDSR1) (Lim et al., 2018) in pepper; and ZmXerico2 in maize (Gao et al., 2012). RING zinc finger proteins located in the nucleus include AL TOLERANCE RING FINGER 1 (AtATRF1) (Qin et al., 2017) and HIGH EXPRESSION OF OSMOTICALLY RESPONSIVE GENES 1 (AtHOS1) (Kim J.-H. et al., 2017) in Arabidopsis; SALT-, ABA- AND DROUGHT-INDUCED RING FINGER PROTEIN 1 (OsSADR1) (Park et al., 2018c) in rice; and ADIP1 INTERACTING RING FINGER PROTEIN 1 (CaAIRF1), CaASRF1, and CaDSR1 (Lim et al., 2017) in pepper.

In addition to the cell membrane, cytoplasm, and nucleus, a few RING zinc finger proteins localize in other subcellular organelles, such as the endoplasmic reticulum, Golgi apparatus, and cytoskeleton. The rice RING-HC zinc finger protein HEAT AND COLD INDUCED 1 (OsHCI1) is mainly distributed near the cytoskeleton (Lim et al., 2013). The rice RING-H2 zinc finger protein OsSIRH2-14 is not only located in the cell membrane and cytoplasm, but also found in the Golgi apparatus (Park et al., 2019). The MICROTUBULE-ASSOCIATED RING FINGER PROTEIN 1 (OsMAR1), a rice RING-H2 zinc finger protein, is found in microtubules (Park et al., 2018b), and the wild tomato RING-H2 zinc finger protein SpRing is distributed in the endoplasmic reticulum (Yang et al., 2016). This shows that RING zinc finger proteins function in numerous biological processes in plants.

## Roles of Ring Zinc Finger Proteins in the Ubiquitin Proteasome Pathway

Ensuring the normal synthesis and degradation of proteins is critical for maintaining growth and development (Marshall and Vierstra, 2019). Proteins are mainly degraded through autophagy and the UPS (Blasiak et al., 2019). Under abiotic stress, large amounts of non-functional and unfolded proteins accumulate in plant cells. The UPS identifies these aberrant proteins and removes them from the cell, which alters the plant proteome and can improve survival under stress conditions (Stone, 2019).

The ubiquitin proteasome pathway has many components, including the 76-amino acid Ub, UBA, UBC, UBL, DUBs, the 26S ubiquitin proteasome, and the target substrate proteins modified by ubiquitination (Liu W. et al., 2020). UPS-mediated protein degradation consists of two stages. First, Ub attaches to the substrate protein, then the ubiquitinated substrate is degraded by the 26S proteasome (Linden and Callis, 2020). The transfer of Ub requires the participation of E1, E2, and E3 enzymes. First, the Cys on the active site of E1 is covalently bound to the terminal Gly of the Ub molecule through a high-energy thioester bond, consuming ATP to activate the Ub. Then the Ub is transferred to the Cys residue of E2 to form an E2-Ub thioester complex, and finally E3 transfers the Ub from E2 to the substrate protein.

As the last enzyme in the first stage of the ubiquitin proteasome pathway, E3 plays a specific role in the recognition of target substrates and then degrades the target protein or changes the activity of the target protein (Swatek et al., 2019). Most RING zinc finger proteins have E3 ubiquitin ligase activity (Joazeiro and Weissman, 2000). E3 ubiquitin ligases are the most diverse component of the UPS, as most plant genomes encode hundreds of E3 ubiquitin ligases, but have many fewer genes encoding other UPS components.

## Roles of Ring Zinc Finger Proteins in Abiotic Stress Responses

**FIGURE 2:**
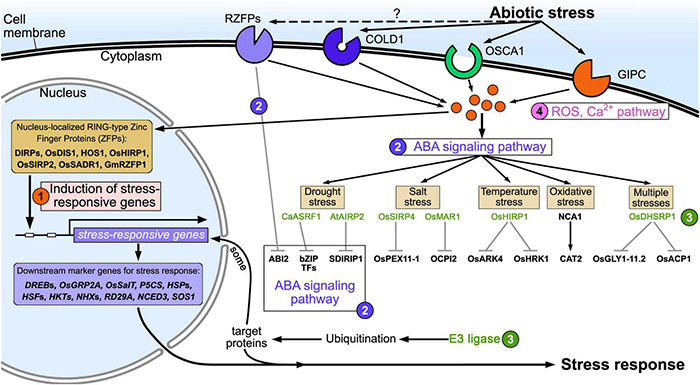
Schematic of RING zinc finger protein regulation of plant adaptation to abiotic stress. ➀ Expression of stress-responsive genes; ➁ ABA pathway; ➂ ubiquitination pathway; ➃ Ca^2+^ and ROS signaling pathway.

Abiotic stress factors include drought, low temperature, high temperature, flooding, saline-alkali soils/water, ROS, and harmful metals. These factors lead to accumulation of misfolded or dysfunctional proteins, which negatively affect the physiological and biochemical processes of plant cells, resulting in reduced growth and production. RING zinc finger proteins respond to a variety of abiotic stresses through different pathways in plants, such as via expression of stress-responsive genes, the ABA pathway, the ubiquitination pathway, ROS and Ca^2+^ signaling (Figure 2).

### Roles in Drought Stress

Drought stress dramatically affects the survival and distribution of plants (Sharma et al., 2018). RING zinc finger proteins respond to drought tolerance by regulating ABA biosynthesis and signal transduction. As a type of isoprenoid phytohormone, the early steps of ABA biosynthesis occur in plastids, and the key enzymes involved in ABA biosynthesis are ZEP, NCED, short chain dehydrogenase/reductase, and ABA aldolase (Vaičiukynė et al., 2019). The three core components of the ABA signaling pathway include ABA receptors such as PYR1, PYL, and RCAR family, PP2Cs (negative regulators), and SnRK2s (positive regulators). In the presence of ABA, the formation of a PYR1/PYL/RCAR-PP2Cs complex results in the inhibition of PP2Cs activity, which in turn activates SnRK2s. The activated SnRK2s then phosphorylate downstream substrate proteins, such as bZIP transcription factors, thereby promoting the transcription of ABA responsive genes (Sah et al., 2016).

### Roles in Abscisic Acid Biosynthesis

RING-H2-type zinc finger proteins in Arabidopsis greatly enhance drought tolerance by increasing ABA synthesis. *AtXERICO* in Arabidopsis encodes a RING-H2-type zinc finger protein. Upon drought stress, *AtNECD3* expression, a key gene in ABA biosynthesis, was rapidly increased in *AtXERICO*-overexpressing Arabidopsis plants compared with the wild type (Ko et al., 2006). When *AtXERICO* was introduced into rice, the drought tolerance of transgenic rice was significantly improved compared to the wild type, and the transpiration and water consumption was lower than that of the wild type. Furthermore, under drought stress, the content of ABA and the expression of four ABA-biosynthesis or ABA-responsive genes, *OsNCED*, *OsABA3*, *OsABI5*, and *OsLEA3-1*, was significantly increased in *AtXERICO*-overexpressing rice compared with the wild type (Zeng et al., 2015). Maize ZmXerico1 and ZmXerico2 (Brugière et al., 2017) are homologs of AtXERICO. Unlike *AtXERICO*, *ZmXerico* genes appear to improve ABA signaling by regulating the stability of ABA 8’-hydroxylase, and ultimately increase the content of ABA in plants. Pepper CaDSR1 (Lim et al., 2018) is a RING-H2-type zinc finger protein with E3 ubiquitin ligase activity. CaDSR1 promotes drought tolerance by degrading the bZIP family protein CaDILZ1, which negatively functions in ABA accumulation.

### Roles in the Abscisic Acid Signal Transduction Pathway

RING zinc finger proteins that function as E3 ubiquitin ligases participate in plant drought tolerance via regulating ABA signal transduction. For example, SALT-AND DROUGHT-INDUCED RING FINGER1 (*SDIR1*) encodes a RING-H2-type E3 ubiquitin ligase and *SDIR1*-overexpressing Arabidopsis lines show ABA hypersensitivity and ABA-related phenotypes during germination, enhanced ABA-induced stomatal closure, and drought tolerance (Zhang et al., 2007), while the *sdir1* loss-of-function mutant exhibits ABA-insensitive phenotypes. Furthermore, complementation with bZIP family genes, such as *ABI5*, *ABF3*, and *ABF4*, can rescue the ABA-insensitive phenotype of the *sdir1* mutant, whereas *SDIR1* overexpression cannot rescue the ABA-insensitive phenotype of an *abi5* loss-of-function mutant. This suggests that SDIR1 acts upstream of some bZIP family transcription factors and may function in the ABA signaling pathway. SDIR1 orthologs in other plant species were found to have similar functions, such as DELAYED SEED GERMINATION 1 (OsDSG1) (Park et al., 2010) and SALT-AND DROUGHT-INDUCED RING FINGER 1 (OsSDIR1) (Gao et al., 2011) in rice, ZmRFP1 in maize (Xia et al., 2012), and ABI3-INTERACTING PROTEIN 2 (AtAIP2) (Zhang et al., 2005) in Arabidopsis. Their target substrates (such as OsABI3 and AtABI3) also share high sequence similarity.

The RING-HC-type zinc finger protein AtAIRP2 (Cho et al., 2011) has E3 ubiquitin ligase activity. *AtAIRP2* is strongly induced by drought and ABA. Compared with wild-type Arabidopsis, the *atairp2* loss-of-function mutant showed reduced sensitivity to ABA and reduced tolerance to drought stress, whereas *AtAIRP2*-overexpression lines showed ABA sensitivity and enhanced drought tolerance. Accordingly, the expression of ABA-induced drought-response genes (such as *RD20*, *RD26*, *RD29A*, and *RD29B*) in the *atairp2* loss-of-function lines was much lower than that in the *AtAIRP2*-overexpression lines. Interestingly, ectopic overexpression of *AtAIRP2* in the *atairp2* mutant rescues the ABA-insensitive and drought-sensitive phenotypes of the mutants. Yeast two-hybrid assays showed that AtAIRP2 interacts with SDIRIP1 (Oh et al., 2017), which is a negative factor in ABA signal transduction. SDIRIP1 selectively regulates the expression of the downstream bZIP transcription factor *ABI5*, rather than *ABF3* or *ABF4*. Further results suggest that SDIRIP1 acts downstream of AtAIRP2, and AtAIRP2 may regulate the Arabidopsis response to ABA and drought stress through ubiquitination of SDIRIP1.

AtAIRP4 (Yang et al., 2016) shows high sequence similarity with AtAIRP2 and also plays a positive role in drought stress. When treated with ABA, the expression of *ABI2* and *ABF3* increased significantly in Arabidopsis lines overexpressing *AtAIRP2*, while there was no significant change in these genes in *AtAIRP4*-overexpression lines. These differences suggest that AtAIRP2 and AtAIRP4 may have different targets and play combined roles in drought stress responses.

The RING-H2 zinc finger proteins RHA2a and RHA2b (Li H. et al., 2011) in Arabidopsis also positively regulate ABA signal transduction and drought stress response. Genetic analyses indicated that RHA2a and RHA2b act as E3 ubiquitin ligases to ubiquitinate downstream substrates of ABI2, thus positively regulating ABA signal transduction. The *rha2a rha2b* double loss-of-function mutant is less sensitive to ABA and more susceptible to drought stress than the single loss-of-function mutants, suggesting that RHA2a functions redundantly with RHA2b in ABA-dependent stress responses.

The pepper RING-HC zinc finger protein CaAIRF1 (Lim et al., 2017) is a RING-type E3 ligase, and positively regulates ABA signaling and drought stress response. CaAIRF1 ubiquitinates and degrades protein phosphatase 1 (CaADIP1), which negatively regulates ABA signaling. Pepper CaASRF1 (Joo et al., 2020) improves the drought tolerance of plants and positively regulates ABA signaling by degrading downstream substrates. As a C3H2C3-type RING E3 ligase, CaASRF1 degrades, via the ubiquitination pathway, the subgroup D bZIP transcription factor CaAIBZ1 and the subgroup A bZIP transcription factor *Capsicum annuum* ASRF1 target bZIP transcription factor 1 (CaATBZ1) (Joo et al., 2019b), which negatively regulate ABA signaling and drought stress response, respectively. Furthermore, a yeast two-hybrid screening for CaATBZ1 identified another RING-type E3 ubiquitin ligase regulator *Capsicum annuum* ATBZ1 Interacting RING finger protein 1 (CaATIR1) (Joo et al., 2020), which mediates CaATBZ1 degradation through the 26S proteasome system.

RING zinc finger proteins are involved in ABA-mediated drought stress tolerance by affecting amino acid transport. AtAIRP3 (Kim J. H. and Kim W. T., 2013) is a RING-HC-type zinc finger protein with E3 ubiquitin ligase activity, and its expression was up-regulated by drought and ABA. The *atairp3* loss-of-function mutant exhibited impaired ABA-mediated seed germination, impaired stomatal closure, and increased sensitivity to drought. *AtAIRP3* is the same gene as *LOG2* (Pratelli et al., 2012), which is involved in amino acid export from the plasma membrane. Two downstream target proteins of AtAIRP3/LOG2, GDU1 and RD21, were identified in a yeast two-hybrid assay. GDU1 is considered to be a subunit of the amino acid output complex on the plasma membrane, and AtAIRP3/LOG2 ubiquitinated GDU1 to stabilize the localization of GDU1 on the plasma membrane. RD21 (Koizumi et al., 1993) is a drought-induced cysteine protease, which may function in re-mobilizing intracellular macromolecules under stress. AtAIRP3/LOG2 ubiquitinated RD21 to prevent excessive degradation of cellular proteins under drought stress. These results suggest that AtAIRP3/LOG2 is not only involved in the ABA-mediated drought stress response, but also functions in amino acid output in Arabidopsis.

RING zinc finger proteins are involved in ABA-mediated drought stress via other mechanisms. The Arabidopsis RING-H2-type zinc finger protein AtAIRP1 (Ryu et al., 2010) has E3 ubiquitin ligase activity. *AtAIRP1*-overexpression lines showed ABA sensitivity and enhanced drought tolerance, whereas the loss-of-function mutants show the opposite phenotypes. The interacting protein of AtAIRP1 is AtKPNB1 (Oh et al., 2020), which is a core component of nuclear transport. AtKPNB1 acts as a negative factor in the ABA-mediated drought stress response. AtAIRP1 regulates the ABA-mediated drought response in Arabidopsis through ubiquitination of AtKPNB1. The wheat RING-HC zinc finger protein TaDIS1 (Liu et al., 2018; Lv et al., 2020) localizes in the Golgi apparatus and has E3 ubiquitin ligase activity. *TaDIS1*-overexpression plants have increased sensitivity to ABA. TaSTP was screened by yeast two-hybrid assays and interacts with TaDIS1 and positively regulates drought tolerance of wheat. OsDIS1 negatively regulated drought tolerance of rice by promoting TaSTP degradation through the 26S proteasome. ABA induced the expression of the RING zinc finger proteins TaRZF70 (Kam et al., 2007), AdZFP1 (Yang et al., 2008), and BdRHP1 (Zeng et al., 2019), which are involved in drought stress. However, their specific molecular mechanisms need further study.

### Roles in Other Signaling Pathways in Drought Stress

In addition to the ABA signaling pathway, RING zinc finger proteins are also involved in drought tolerance through the MAPK signaling pathway. RGLG1 and RGLG2, as E3 ubiquitin ligases, ubiquitinate and degrade MAPKKK18, a mitogen-activated protein kinase, to decrease drought tolerance (Yu et al., 2021).

RING zinc finger proteins also function in drought tolerance by regulating ROS content. AtATL78, located in the plasma membrane, is a RING-H2-type E3 ubiquitin ligase in Arabidopsis (Kim S. J. and Kim W. T., 2013). *atatl78* loss-of-function mutants showed decreased tolerance to drought, while *AtATL78* overexpression produced the opposite phenotype. Studies found that loss of AtATL78 led to a decrease of CAT and H_2_O_2_. AtATL78 may promote ROS-mediated ABA signaling to improve drought tolerance (Suh et al., 2016). The potato RING zinc finger StRFP2 (Qi et al., 2020) is an E3 ubiquitin ligase, which enhanced drought tolerance by increasing the free proline content in potato and CAT activity in *StRFP2*-overexpressing *N. benthamiana* lines.

RING zinc finger proteins can also improve drought tolerance by regulating the content of osmoregulatory substances. AtRZF1 (Ju et al., 2013) is a RING-H2-type zinc finger protein with E3 ubiquitin ligase activity. The proline content was higher in the Arabidopsis *atrzf1* loss-of-function mutant than that in the wild type and *AtRZF1*-overexpression plants. In subsequent experiments, *atrzf1* mutants were used as materials for T-DNA tag insertion. After screening, three double mutants, *atrzf1 pcp17*, (Shin et al., 2019) *atrzf1 pca21* (Park et al., 2017), and *atrzf1 pca22* (Kim A.-R. et al., 2017), were found to have reduced proline content compared with the *atrzf1* single mutant under drought conditions. These three mutations inhibited the drought-insensitive phenotype of the *atrzf1* mutant by reducing the proline content, suggesting that AtRZF1 may function through the proline pathway.

RING zinc finger proteins also improve drought tolerance by mediating the ubiquitination of drought stress-related proteins. DREB2A-INTERACTING PROTEIN1 (DRIP1) and DRIP2 (Qin et al., 2008) are both RING-HC-type zinc finger proteins with E3 ubiquitin ligase activity in Arabidopsis. Drought-related gene expression was significantly enhanced in the *drip1 drip2* double loss-of-function mutant under drought stress. *In vitro* ubiquitination experiments showed that DRIP1 and DRIP2 mediate the ubiquitination of DREB2A, thereby negatively regulating plant tolerance to drought stress. The RING-HC zinc finger protein OsDIS1 (Ning et al., 2011a,b) functions as an E3 ubiquitin ligase to negatively regulate drought tolerance in rice by promoting OsNek6 and OsSKIPa degradation. RGLG2 (Cheng et al., 2012) translocates from the plasma membrane to the nucleus and interacts with AtERF53 under drought stress. RGLG2 plays a negative role in resisting drought stress by degrading AtERF53 through the ubiquitination pathway. The *N. benthamiana* RING-H2 zinc finger protein NtRHF1 (Xia et al., 2013) is an ortholog of AtSDIR1. AtUAP1 (Min et al., 2021) interacted with AtRZF1. Ubiquitin-Associated Protein 1 (AtUAP1) contains a Ub-associated motif, and *AtUAP1*-overexpressing Arabidopsis plants were sensitive to drought stress, but the *atrzf1* mutation rescued this phenotype. This suggests that AtRZF1 may also participate in the Arabidopsis response to drought stress through AtUAP1 ubiquitination.

RING zinc finger proteins can also improve drought tolerance through the endoplasmic reticulum stress pathway. AtRMA1 in Arabidopsis (Matsuda et al., 2001) and CaRma1H1 in pepper (Lee et al., 2009) are both RING-HC-type zinc finger proteins with E3 ubiquitin ligase activity. AtRma1 and CaRma1H1 are both located on the endoplasmic reticulum, both are induced by abiotic stress, and both inhibit the transport of the aquaporin PIP2;1 from the endoplasmic reticulum to the plasma membrane through ubiquitination. However, there is also an interesting difference between AtRma1 and CaRma1H1; *CaRma1H1* overexpression in Arabidopsis is much more stable than *AtRma1* overexpression in Arabidopsis, which explains why Arabidopsis *CaRma1H1*-overexpression lines have enhanced drought tolerance and Arabidopsis *AtRMA1*-overexpression lines show no obvious phenotypic changes compared to wild-type Arabidopsis. Overexpression of OsRDCP1 (Bae et al., 2011), a homolog of CaRma1H1, in rice also enhanced drought tolerance. Furthermore, overexpression of *CaRma1H1* in cultivated tomato (*Solanum lycopersicum*) also enhanced drought tolerance (Seo et al., 2012), and the expression of downstream endoplasmic reticulum stress marker genes (such as *LeCNX1*, *LeBIP1*, and *LePDIL1*) was significantly up-regulated in the transgenic tomato, suggesting that CaRma1H1 overexpression may enhance drought tolerance not only by regulating aquaporins but also by modulating endoplasmic reticulum stress.

### Roles in Temperature Stress

Extreme temperatures (too low or too high) affect plant metabolic activities, cell membrane fluidity, membrane lipid composition, photosynthesis, and respiration, thus affecting plant growth and development (Ding et al., 2020). RING zinc finger proteins are involved in the response to temperature stress.

### Roles in Low Temperature Stress

RING zinc finger proteins are involved in the response to low temperature by ubiquitinating low temperature-induced transcription factors. The RING-HC zinc finger protein HIGH EXPRESSION OF OSMOTICALLY RESPONSIVE GENES 1 (HOS1) (Dong et al., 2006) with E3 ubiquitin ligase activity in Arabidopsis is a key negative regulator of plant tolerance to low temperature. HOS1 interacted with ICE1 to mediate ICE1 ubiquitination and degradation. Therefore, *HOS1* overexpression inhibited the expression of CBF genes and decreased tolerance to low temperature in Arabidopsis.

RING zinc finger proteins can improve the low temperature tolerance of plants by increasing the content of osmoregulation substances. In rice, the RING-H2 zinc finger protein COLD-INDUCIBLE (OsCOIN) (Liu et al., 2007) is induced by low temperature. *OsCOIN* overexpression in transgenic rice not only significantly improved cold tolerance, but also increased *OsP5CS* expression and increased the proline content, which plays an important role in cell osmotic balance.

### Roles in High Temperature Stress

In addition to being responsive to low temperatures, RING zinc finger proteins are also involved in the response to high temperature.

RING zinc finger proteins participate in high temperature tolerance by activating DNA repair systems. Heat tolerance and DNA repair capacity were significantly reduced in the *HOS1*-deficient Arabidopsis mutants, in which heat-induced expression of DNA repair system genes, such as the DNA helicase *RECQ2*, was significantly reduced (Han et al., 2020c). Furthermore, HOS1 is heat stable in a heat shock factor Hsp90-dependent manner. The heat-responsive HSP90-HOS1-RECQ2 module helps maintain genome integrity under high temperature stress, demonstrating a significant relationship between DNA repair and plant heat tolerance.

RING zinc finger proteins can ubiquitinate high-temperature-tolerance-related proteins by trafficking between different subcellular organelles under heat stress. The rice RING-HC zinc finger protein HEAT AND COLD INDUCED 1 (OsHCI1) (Lim et al., 2013) is induced by high temperature. OsHCI1 mainly localizes to the Golgi apparatus and moved rapidly and widely in the cytoskeleton. At high temperature, OsHCI1 accumulates in the nucleus. Heat shock-induced accumulation of OsHCI1 mediates nucleocytoplasmic trafficking of nuclear substrate proteins through monoubiquitination, thereby acting as an inactivation mechanism to improve heat tolerance. Furthermore, the sorghum (*Sorghum bicolor*) ortholog of OsHCI1, SbHCI1 (Lim et al., 2020), is also a RING E3 ubiquitin ligase, and SbHCI1 predominantly localizes to the cytosol, but it moves to the Golgi under heat stress. Ectopic overexpression of *SbHCI1* in Arabidopsis improved ABA sensitivity and heat stress tolerance. Consistent with OsHCI1, SbHCI1 also acts as a positive regulator of the heat stress response.

RING zinc finger proteins improve high temperature tolerance by ubiquitinating high-temperature-tolerance-related proteins. Rice HEAT-INDUCED RING FINGER PROTEIN 1 (OsHIRP1) (Kim et al., 2019) is a RING-HC zinc finger protein with E3 ligase activity, which positively regulates the response to high temperature stress. Two OsHIRP1-interacting proteins, OsARK4 and OsHRK1, were screened and identified by yeast two-hybrid and BiFC assays. *In vitro* ubiquitination assays revealed that OsHIRP1 directly ubiquitinates OsARK4 and OsHRK1. In addition, under heat stress, heat stress-inducible genes, such as *HsfA3*, *HSP17.3*, *HSP18.2*, and *HSP20*, were up-regulated in *OsHIRP1*-overexpressing Arabidopsis lines compared to the control.

RING zinc finger proteins are involved in high temperature tolerance through the ABA signaling pathway. The rice RING-HC zinc finger protein OsRZFP34 (Hsu et al., 2014) is an E3 ubiquitin ligase, and its expression is induced by high temperature and ABA treatment. The relative stomatal opening was increased in rice and Arabidopsis plants overexpressing *OsRZFP34* compared with their wild types. The analysis of water loss and leaf temperature showed that the evaporation rate and cooling effects of *OsRZFP34* overexpression in rice and Arabidopsis were higher than those of their wild types, and *OsRZFP34* expression had protective effects on stomatal opening, leaf cooling, and tolerance to high temperature stress. The rice RING-H2 zinc finger protein HEAT TOLERANCE AT SEEDLING STAGE (OsHTAS) (Liu et al., 2016) is an E3 ubiquitin ligase. OsHTAS regulates H_2_O_2_ accumulation in leaves, alters the stomatal pore size of leaves, and promotes ABA biosynthesis. OsHTAS enhances heat tolerance by regulating stomatal closure induced by H_2_O_2_, and participates in drought and salt tolerance pathways through the ABA signaling pathway.

RING zinc finger proteins are also involved in high temperature tolerance through the ethylene signaling pathway. Ethylene, a gaseous phytohormone, plays a crucial role in plant adaptive responses to temperature changes. As an E3 ubiquitin ligase, SALT-AND DROUGHT-INDUCED RING FINGER1 (SDIR1) (Hao et al., 2021) positively regulates the ethylene response. SDIR1 mediates temperature-induced EBF1/EBF2 degradation and EIN3 accumulation to fine tune the ethylene response to environmental temperature changes, enabling plants to adapt to fluctuating environmental conditions.

### Roles in Salt Stress

Long-term exposure to high salinity results in a high osmotic potential and ion toxicity within the plant, which inhibits growth and development (Acosta-Motos et al., 2017). The expression of genes encoding RING zinc finger proteins was up-regulated under high salt conditions (Dos Reis et al., 2012). Many RING zinc finger proteins have a potential role in salt stress tolerance.

RING zinc finger proteins improve salt stress tolerance by regulating ion homeostasis and ROS scavenging. Arabidopsis SALT TOLERANCE RING FINGER1 (AtSTRF1) (Tian et al., 2015) is a RING-H2-type zinc finger protein. AtSTRF1 has E3 ubiquitin ligase activity *in vitro*, and *STRF1*-overexpressing Arabidopsis lines exhibit enhanced tolerance to salt and osmotic stresses, reduced ROS accumulation under salt stress, and increased expression of *AtRbohD*, which encodes a NADPH oxidase involved in H_2_O_2_ production. STRF1 is a ubiquitin ligase related to membrane transport, which helps plants respond to salt stress by monitoring intracellular membrane transport and ROS production. The rice RING-H2-type zinc finger protein OsSIRH2-14 (Park et al., 2019) has E3 ubiquitin ligase activity and functions in salt tolerance by modifying salt stress-related proteins such as OsHKT2. *OsSIRH2-14* is expressed in NaCl-treated root and stem tissues. Compared with wild-type plants, *OsSIRH2-14*-overexpressing rice plants show obvious salt tolerance, and the accumulation of Na^+^ in the aboveground and root tissues is significantly reduced. *In vitro* pull-down experiments and BiFC showed that OsSIRH2-14 interacted with salt stress-related proteins, including OsHKT2;1, OsSalT, and OsPRF2. OsSIRH2-14 may positively regulate the salt stress response by regulating the stability of salt-related proteins. Rice RING FINGER PROTEIN V6 (OsRFPv6) (Kim et al., 2021) is a RING-HC-type zinc finger protein with E3 ubiquitin ligase activity that positively regulates salt tolerance in rice. *OsRFPv6* was highly expressed under saline conditions. Under salt stress, *OsRFPv6*-overexpression plants were much less sensitive to salt stress compared with wild-type rice, and *OsRFPv6*-overexpression lines had higher levels of proline, soluble sugars, and chlorophyll, and lower H_2_O_2_ accumulation than wild-type rice. The expression of the Na^+^ transporter genes *OsHKT2;1* and *OsCNGC1* was significantly lower in the roots and shoots of the *OsRFPv6*-overexpression plants than in the wild-type plants under salt stress. However, *OsHKT1;5*, which encodes a transporter involved in phloem Na^+^ exclusion to prevent leaf Na^+^ accumulation, was significantly up-regulated in roots and shoots of the *OsRFPv6*-overexpression plants. Furthermore, expression of the genes encoding the Na^+^/H^+^ antiporter OsNHX1, located in the vacuolar membrane, the Na^+^/H^+^ antiporter OsSOS1, located in the plasma membrane, and the K^+^ transporter OsHAK7 was significantly up-regulated in the roots and shoots of the *OsRFPv6*-overexpression plants. These results suggest that OsRFPv6 positively regulates salt stress responses by regulating the expression of Na^+^-related transporter genes. Rice OsSIRP1 (Hwang et al., 2016) is a RING-HC-type zinc finger protein with E3 ubiquitin ligase activity. *OsSIRP1*-overexpressing Arabidopsis lines had attenuated salt tolerance during seed germination and root growth. Consistent with OsSIRP1, *OsSIRP4*-overexpression plants also exhibited hypersensitivity to salt responses (Kim and Jang, 2021). Yeast two-hybrid and BiFC analyses showed that OsSIRP4 regulates the degradation of its substrate PEROXISOMAL BIOGENESIS FACTOR 11-1 (OsPEX11-1) through the 26S proteasome system. OsPEX11 is involved in salt stress tolerance by regulating the expression of cation transporter and antioxidant defense genes, and can significantly improve the salt tolerance of rice (Cui et al., 2016). In *OsSIRP4*-overexpressing plants, the enzymatic activities of CAT, SOD, and APX were significantly reduced, and the accumulation of soluble sugars and proline also decreased, but the H_2_O_2_ content was significantly elevated. Furthermore, qRT–PCR showed that the expression of Na^+^/K^+^ homeostasis-related genes *AtSOS1*, *AtAKT1*, *AtNHX1*, and *AtHKT* decreased in the transgenic plants. These results indicate that OsSIRP4 regulates OsPEX11-1 accumulation through the ubiquitination pathway, and OsPEX11-1 most likely regulates its downstream genes (such as *AtSOS1*, *AtAKT1*, *AtNHX1*, and *AtHKT*) in response to salt stress. The sweet potato (*Ipomoea batatas*) RING-H2-type zinc finger protein IbATL38 (Du et al., 2021) is an E3 ubiquitin ligase. *IbATL38* expression was strongly induced by NaCl and ABA. Subcellular localization showed that IbATL38 localized in the nucleus and plasma membrane. Arabidopsis lines overexpressing *IbATL38* exhibited enhanced salt tolerance and reduced H_2_O_2_ content. Under salt stress, the upregulation of ROS scavenging system genes (such as *AtSOD*, *AtPOD*, and *AtAPX*) in *IbATL38*-overexpressing Arabidopsis lines was much higher than that in wild-type Arabidopsis. Therefore, the ROS content in the overexpression lines was lower than that in wild-type Arabidopsis under salt stress. These results suggest that IbATL38 may improve plant tolerance to salt stress by promoting ROS scavenging.

RING zinc finger proteins improve plant salt tolerance via the ABA signaling pathway. The Arabidopsis RING-H2-type RING zinc finger protein SDIR1 (Zhang et al., 2015) is an E3 ligase. *SDIR1* expression is up-regulated under salt stress. *SDIR1* overexpression leads to increased ABA sensitivity and phenotypic changes related to ABA signaling pathways, such as increased salt sensitivity during germination, ABA-induced stomatal closure, and enhanced drought tolerance. SDIR1 targets SDIRIP1 for degradation to modulate the salt stress response and ABA signaling in Arabidopsis. Wild tomato *SpRing* (Qi et al., 2016) encodes a RING-H2-type zinc finger protein with E3 ubiquitin ligase activity. *SpRing* is expressed in all tissues of wild tomato and its expression is regulated by salt stress. Virus-induced gene silencing lines showed increased sensitivity to salt stress, while *SpRing*-overexpressing transgenic Arabidopsis showed enhanced salt tolerance during seed germination and early seedling development. Under salt stress, the Na^+^ content in the shoots of *SpRing*-silenced tomato plants was much higher than that in the control group. Further, expression of the ABA-independent gene *SlRD29A* and the ABA-dependent gene *SlNCED* was decreased in *SpRing*-silenced tomato plants, which was consistent with the increased expression of *AtRD29A* and *AtNCED3* in *SpRing*-overexpressing Arabidopsis plants. These results indicate that SpRing is involved in ion homeostasis under salt stress and may play a positive regulatory role in both ABA-dependent and ABA-independent pathways.

RING zinc finger proteins improve plant salt tolerance by ubiquitinating salt tolerance-related proteins. Rice plants overexpressing the RING-H2-type zinc finger gene *OsSIRP3* (Park et al., 2018d) showed hypersensitive phenotypes to salt stress including reduced seed germination rate and root growth. OsSIRP3 interacted with the salt-induced proteins OsMADS70 and OsABC1P11 in a yeast two-hybrid assay. Therefore, OsSIRP3 may negatively regulate salt stress responses by regulating these two proteins. However, unlike OsSIRP1, OsSIRP3, and OsSIRP4, the RING-HC zinc finger protein OsSIRP2 (Chapagain et al., 2018) showed a positive regulatory effect on the salt stress response. Heterologous overexpression of *OsSIRP2* in Arabidopsis plants resulted in enhanced tolerance to salt and osmotic stresses. Rice TRANSKETOLASE1 (OsTKL1) was identified as an OsSIRP2-interacting partner by yeast two-hybrid assay. Transketolases play different roles in plant growth, development, and physiology (Bernacchia et al., 1995). This finding indicates that OsSIRP2 may positively regulate the plant response to salt and osmotic stresses by regulating OsTKL1. The rice RING-H2-type zinc finger protein OsMAR1 (Park et al., 2018b) with E3 ligase activity is highly expressed under high salinity. Compared with the wild type, *OsMAR1*-overexpressing Arabidopsis lines showed slow root growth and exhibited hypersensitivity under high salinity. Yeast two-hybrid and BiFC assays indicated that OsMAR1 interacts with rice CHYMOTRYPSIN INHIBITOR 2 (OsCPI2) and degrades OsCPI2 through the ubiquitination pathway. While heterologous overexpression of *OsCPI2* leads to enhanced growth and enhanced salinity and osmotic stress tolerance in transgenic Arabidopsis (Tiwari et al., 2015), OsMAR1 may negatively regulate the salt stress response by regulating OsCPI2 in rice.

### Roles in Oxidative Stress

Under oxidative stress, the metabolic homeostasis of active oxygen is destroyed, resulting in cellular ROS accumulation (Nadarajah, 2020). High concentrations of superoxide radicals, hydroxyl radicals, and H_2_O_2_ oxidatively damage biological macromolecules, such as protein and DNA, and affect plant growth and development (Pardo-Hernández et al., 2020). Some RING zinc finger proteins are also involved in oxidative stress responses in plants.

Soybean GmRZFP1 (Wu et al., 2010) is a RING-HC-type zinc finger protein involved in oxidative stress in plants. Under oxidative stress, compared with wild-type soybean, *GmRZFP1*-overexpressing soybean lines had stronger tolerance to oxidative stress. Arabidopsis AtOHRP1 (Li et al., 2013) is also a HC-type RING zinc finger protein, and *AtOHRP* expression is significantly induced by H_2_O_2_. Analysis of changes of the H_2_O_2_ content and antioxidant enzyme activity in plants after oxidative stress revealed that the *atohrp* mutant accumulated more H_2_O_2_, and the activities of key enzymes in the active oxygen scavenging system, such as SOD, CAT, and APX, decreased significantly compared with the wild type. This indicated that AtOHRP1 has an important physiological function in oxidative stress. Arabidopsis AtNCA1 (Li J. et al., 2015), a RING-type zinc finger protein, interacts with and activates CAT, and maintains the proper CAT folding to ensure maximum CAT enzyme activity under oxidative stress, to maintain ROS homeostasis, and to enhance the tolerance to oxidative stress. Arabidopsis lines with mutated NCA1 are hypersensitive to multiple abiotic stresses.

Apple MdMIEL1 (An et al., 2017b) is a RING zinc finger protein with E3 ubiquitin ligase activity. *MdMIEL1* expression was significantly induced by H_2_O_2_. *MdMIEL1*-overexpressing Arabidopsis and apple calli exhibited higher H_2_O_2_ sensitivity compared to the wild-type controls. DAB and NBT tissue staining showed that *MdMIEL1*-overexpressing plants exhibited more ROS accumulation than wild-type plants without H_2_O_2_ treatment, suggesting that MdMIEL1 may be involved in ROS homeostasis. Yeast two-hybrid assay identified MdMYB1 as the interactor of MdMIEL1 (An et al., 2017a). MdMYB1 regulates anthocyanin biosynthesis in apple, and anthocyanins are closely associated with various abiotic stresses such as oxidative stress. MdMIEL1 negatively regulates anthocyanin accumulation by degrading MdMYB1 through the ubiquitination pathway. MdMIEL1 negatively regulates oxidative stress tolerance by regulating ROS homeostasis and anthocyanin biosynthesis. Further studies showed that MdMIEL1 also regulates lateral root initiation by increasing auxin accumulation in *MdMIEL1*-overexpressing Arabidopsis roots (An et al., 2017b).

### Roles in Harmful Metal Stress

Harmful metals such as cadmium and aluminum can substantially hinder plant growth and development. Heavy metal cadmium accumulation in plants seriously affects the absorption and transportation of nutrients and water, increases oxidative damage, perturbs metabolism, and ultimately inhibits plant growth and development (Haider et al., 2021). Some 50% of the world’s potential arable land is acidic soil (Chauhan et al., 2021), and light metal aluminum ions have become the main factor limiting plant growth in acidic soil by inhibiting root growth, increasing oxidative stress, inhibiting photosynthesis, and interfering with the absorption and transportation of water and nutrients (Sade et al., 2016). Traditional methods such as fertilizer application and physical modification of soil composition to help plants cope with cadmium and aluminum stress have shown limited success.

RING zinc finger proteins enhance harmful metal tolerance by reducing the accumulation of metals and alleviating oxidative stress. The soybean RBR type zinc finger protein GmARI1 (Biniek et al., 2017) enhances plant tolerance to aluminum stress. GmARI1 is an E3 ubiquitin ligase. qRT-PCR showed that *GmARI1* expression in soybean roots was strongly induced by aluminum, and *GmARI1*-overexpressing Arabidopsis plants exhibited significantly enhanced aluminum stress tolerance compared with wild-type Arabidopsis. *Glyma02g15070* was the top gene co-expressed with *GmARI1*, and the *Glyma02g15070* ortholog in Arabidopsis, *AT1G49670*, is involved in oxidative stress tolerance (Rounds and Larsen, 2008). SlRING1 (Ahammed et al., 2020) is a RING E3 ligase that positively regulates cadmium tolerance in cultivated tomato. When cadmium stress was encountered, compared with the wild type, *SlRING1*-overexpressing tomato lines not only had an increased chlorophyll content, net photosynthetic rate, and maximal photochemical efficiency of photosystem II, but also decreased ROS levels and relative electrolyte leakage. Furthermore, the improvement of cadmium tolerance in *SlRING1*-overexpression plants was also suggested to be associated with the increased transcript levels of CAT, DHAR, MDHAR, GSH, and PCS genes, which are involved in antioxidant and detoxification systems. More importantly, *SlRING1*-overexpression plants also had decreased cadmium concentrations in shoots and roots compared to wild-type tomato.

RING zinc finger proteins can temporarily ameliorate the DNA damage caused by harmful metal stress through the ubiquitination pathway. The Arabidopsis RING-RH-type zinc finger protein AL TOLERANCE RING FINGER 1 (AtATRF1) (Qin et al., 2017) also enhances plant tolerance to aluminum stress. Like *GmARI1*, *AtATRF1* also encodes an E3 ubiquitin ligase, and its expression is increased by aluminum treatment. *AtATRF1*-overexpressing Arabidopsis lines showed enhanced tolerance to aluminum compared with wild-type plants. Curiously, the expression of nine genes related to aluminum stress tolerance showed no significant differences between wild-type plants and *AtATRF1*-overexpressing lines. However, compared with wild-type plants, the expression of *AtATR* (a gene that monitors DNA integrity and controls cell cycle progression) (Rounds and Larsen, 2008) was lower in *AtATRF1*-overexpressing plants.

## Mechanisms of Ring Zinc Finger Proteins in Abiotic Stress

When plants face abiotic stresses such as drought, high salt, high or low temperature, and heavy metals, these external stress signals are first sensed by the receptors or sensors on the cell’s plasma membrane, and are transmitted to the cell through ROS or Ca^2+^ signals (Choi et al., 2017; Marcec et al., 2019; Chen and Yang, 2020). Therefore, abiotic stress receptors on the plasma membrane play a crucial role in the plant’s response to stress. However, our knowledge of abiotic stress receptors on plant cell plasma membranes is limited, and only a few receptors have been reported, such as low temperature receptors (COLD1) (Ma et al., 2015), drought stress receptors (OSCA1) (Yuan et al., 2014), and salt stress receptors (GIPC) (Jiang et al., 2019; Figure 2). Numerous studies have found that RING zinc finger proteins are significantly induced by drought (Joo et al., 2020; Yu et al., 2021), high salt (Liu Y. et al., 2020; Kim and Jang, 2021), high or low temperature (Han et al., 2020c; Lim et al., 2020), and heavy metals (Qin et al., 2017; Ahammed et al., 2020). This indicates that RING zinc finger proteins participate in the response to abiotic stresses either directly or indirectly. Interestingly, subcellular localization found that some RING zinc finger proteins accumulate on the cell plasma membrane and are transmembrane proteins (Cheng et al., 2012; Suh et al., 2016). For example, AtATL78 is involved in cold and drought stress (Suh et al., 2016), RHA2a (Li H. et al., 2011) and OsRDCP1 (Bae et al., 2011) are involved in drought stress, and OsRFPv6 is involved in salt stress (Kim et al., 2021). Bioinformatics analysis found that these transmembrane proteins have an extracellular domain, transmembrane domain, and intracellular domain. The extracellular domains of some RING zinc finger proteins are negatively charged (such as AtATL78, RHA2a, and OsRFPv6), and some are positively charged (such as OsRDCP1), as shown in Figure 1. These charged extracellular domains may react with harmful stress-related ions such as Na^+^ or Cl^–^ in the response to the external abiotic stress, and thus act as sensors in stress perception. The intracellular domains transmit these abiotic stress signals to the cytoplasm. For example, AtATL78 may promote ROS signal-mediated ABA signaling to improve plant stress tolerance (Suh et al., 2016); the extracellular domain of OsRFPv6 may be involved in salt stress (Kim et al., 2021); and RHA2a mediates drought stress directly through ubiquitinating ABI2 in the ABA receptor signaling pathway. These cases indicate that RING zinc finger proteins on the plasma membrane may function as sensors or ABA receptors in abiotic stress signaling, which needs further study. The amino acid sequence of RING zinc finger proteins expressed on the extracellular plasma membrane is shown in Supplementary Table 2.

In addition, some RING zinc finger proteins accumulate in the nucleus (such as DIRPs, OsDIS1, HOS1, and OsHIRP1). Under abiotic stress, these RING zinc finger proteins may act like transcription factors to regulate the expression of downstream abiotic stress marker genes (such as *DREBs*, *HSPs*, *HKTs*, and *RD29A*) through direct or indirect ways (Qin et al., 2008; Ning et al., 2011a; Kim et al., 2019; Han et al., 2020c).

RING zinc finger proteins usually accumulate in the cytoplasm or nucleus and act as E3 ubiquitin ligases (such as CaASRF1 and AtAIRP2) in the abiotic stress response through ABA, MAPK, and ethylene signaling pathways. RING zinc finger proteins play a key role in ABA signaling especially under drought conditions. They have positive or negative roles in stress tolerance through ubiquitination and degradation of ABA receptor and ABA synthesis-related proteins (such as bZIP transcription factors and SDIRIP1) (Li J. et al., 2015; Oh et al., 2017; Joo et al., 2019a). In addition, a few RING zinc finger proteins, such as NCA1 (Li J. et al., 2015), interact with and activate target proteins involved in stress tolerance (such as CAT2) to maintain correct folding and functionality of the target protein.

The possible mechanisms of RING zinc finger proteins in plant abiotic stress tolerance are shown in Figure 2, and the expression patterns, resistance manners, and references of RING zinc finger proteins related to abiotic stress are summarized in Supplementary Table 3.

## Summary and Future Prospects

The growth and development of plants and their ability to adapt to various stresses are mainly achieved by protein modifications and altering metabolic pathways. Studying the expression and function of RING zinc finger proteins is of great importance to improve plant growth, development, and abiotic stress tolerance.

Protein ubiquitination is one of the most important post-translational modifications of plant proteins. Most RING zinc finger proteins function as E3 ligases in different cellular processes via degrading or modifying their substrate proteins through the 26S proteasome. Ubiquitin E3 ligase determines the specific substrate proteins. However, how E3 ligases interact with substrate proteins, whether they recognize substrate proteins with the same characteristics, and whether they can modify substrate proteins through ubiquitin or polyubiquitin remains to be further determined. The ubiquitin pathway involves many types of RING finger proteins, and each type of RING finger domain may correspond to a variety of E2 ubiquitin binding enzymes. Therefore, further biochemical analysis is needed to combine E2 ubiquitin binding enzymes from different plants with E3 enzymes to further understand how E2-E3 enzymes interact.

Further analysis of the mechanisms of action of RING zinc finger proteins is needed to answer the above questions. New biotechnological tools will also accelerate the research on RING zinc finger proteins. For example, bioinformatics analysis combined with functional genomics and proteomics can be used to infer the corresponding DNA sequences from amino acid sequences of unknown RING zinc finger proteins. The function of unknown RING zinc finger proteins could be identified by gene knockout, gene silencing, or gene overexpression approaches. Additionally, single cell sequencing, spatial transcriptomics, and metabolomics can be used to gain further insight into their structures and functions. Furthermore, the structure and function of RING zinc finger proteins can be artificially changed via clustered regularly interspaced short palindromic repeats (CRISPR)/CRISPR-associated protein 9 (Cas9) genome editing.

Although RING zinc finger proteins have been studied in many species, the current research has mainly focused on Arabidopsis and rice, while little is known about these proteins in other species, especially in stress-resilient plants. Therefore, the function of RING zinc finger proteins still needs to be discovered and studied in halophytes, xerophytes, and heavy metal-tolerant plants.

In addition, the RING zinc finger proteins that are associated with the plasma membrane warrant further study. Plants sometimes have cross adaptation to multiple stresses, and one stress may have multiple effects. For example, salt stress can lead to osmotic stress and ionic stress. Identifying receptors of abiotic stress signals in plants is a hotspot of research and is difficult to study. Studying the characteristics of extracellular domains of transmembrane RING zinc finger proteins could help to clarify their functions in stress signaling. While some transmembrane RING zinc finger proteins (such as AtATL78, RHA2a, and OsRDCP1) with a charged extracellular domain have only been reported to be involved in drought stress, they may also participate in salt and/or heavy metal stresses. Therefore, molecular biological techniques, such as CRISPR-Cas9, for domain modification could help to determine how plasma membrane-localized RING zinc finger proteins perceive abiotic stress signals.

Another important finding is that some RING-type zinc finger proteins undergo nucleocytoplasmic shuttle under stress like CCCH zinc finger proteins (Han et al., 2021). For example, OsHIRP1 has an NLS sequence and should theoretically be located in the nucleus, but at room temperature, 100% of rice protoplasts showed cytoplasmic and nuclear localization. However, under heat treatment (38°C), 11.1% of rice protoplasts showed only nuclear localization, and 88.9% of rice protoplasts still showed cytoplasmic and nuclear localization. Interestingly, under severe heat stress (45°C), 95.8% of rice protoplasts showed nuclear localization, and only 4.2% showed cytoplasmic and nuclear localization (Kim et al., 2019). The mechanism and significance of nucleocytoplasmic shuttle of these RING zinc finger proteins need to be further studied.

Overall, RING zinc finger proteins have great potential in plant stress tolerance. Genetic engineering of RING zinc finger proteins may provide a useful approach to accelerate crop stress tolerance breeding and thus promote sustainable agriculture.

## Author Contributions

GH and ZQ wrote this manuscript. YL, ZY, CW, YZ, and LL participated in the writing and modification of this manuscript. GH and BW conceptualized the idea. All authors read and approved the final manuscript.

## Conflict of Interest

The authors declare that the research was conducted in the absence of any commercial or financial relationships that could be construed as a potential conflict of interest.

## Publisher’s Note

All claims expressed in this article are solely those of the authors and do not necessarily represent those of their affiliated organizations, or those of the publisher, the editors and the reviewers. Any product that may be evaluated in this article, or claim that may be made by its manufacturer, is not guaranteed or endorsed by the publisher.

## Abbreviations

ABA: abscisic acid
MAPK: mitogen-activated protein kinase
ROS: reactive oxygen species
UPS: ubiquitin proteasome system
Ub: ubiquitin molecules
UBA: ubiquitin activating enzyme E1
UBC: ubiquitin conjugating enzyme E2
UBL: ubiquitin ligase E3
DUBs: deubiquitinating enzymes
Cys: cysteine residue
Gly: glycine
ZEP: zeaxanthin epoxidase
NCED: 9-*cis*-epoxycarotenoid dioxygenase
PYR1: ACTIN RESISTANCE 1
PYL: PYR1-LIKE
RCAR: REGULATORY COMPONENTS OF ABA RECEPTORS
PP2Cs: type 2C protein phosphatases
SnRK2s: SNF1-related protein kinase 2s
bZIP: basic region/leucine zipper
H_2_O_2_: hydrogen peroxide
CAT: catalase
SOD: superoxide dismutase
APX: ascorbate peroxidase
POD: guaiacol peroxidase
CBF: C-repeat binding factor
Hsp90: A1/heat shock protein 90
BiFC: biomolecular fluorescence complementation
NADPH: nicotinamide adenine dinucleotide phosphate
RBR: Ring-Between-Ring fingers
qRT-PCR: quantitative real-time polymerase chain reaction
DHAR: dehydroethrin reductase
MDHAR: monodehydroascorbate reductase
GSH: glutathione
PCS: phytochelatin synthase
GST: glutathione *S*-transferase
COLD1: CHILLING-TOLERANCE DIVERGENCE 1
OSCA1: reduced hyperosmolality-induced [Ca^2+^]_i_ increase 1
GIPC: glycosyl inositol phosphorylceramide
DAB: 3,3 ′ -diaminobenzidine
NBT: nitro blue tetrazolium.
