# Supplementary material for: RING Zinc Finger Proteins in Plant Abiotic Stress Tolerance
Source: Front Plant Sci. 2022 Apr 14;13:877011. doi: 10.3389/fpls.2022.877011 (PMC9047180; doi:10.3389/fpls.2022.877011)
Supplement: Supplementary file 1 [file Table_1.DOCX]

**Supplementary Table 1** The type and number of different domains of RING zinc finger proteins in different species

| Species | Different domains | | | | | | | | | | | | | | | Reference |
| --- | --- | --- | --- | --- | --- | --- | --- | --- | --- | --- | --- | --- | --- | --- | --- | --- |
|  | RING-H2 | RING-HC | RING-HCa | RING-HCb | RING-v | RING-C2 | RING-D | RING-S/T | RING-G | RING-mh2 | RING-mhc | C2SHC4 | C3GC3S | C2HC5 | C3HCHC2 |  |
| *Arabidopsis thaliana* | 197 | 258 |  |  | 26 | 16 | 7 | 3 | 1 |  |  |  |  |  |  | ([Jiménez-López et al., 2018](#_ENREF_3)) |
| *Brassica rapa* | 215 | 371 |  | 47 | 44 | 38 | 10 | 5 | 1 |  |  |  |  |  |  | ([Alam et al., 2017](#_ENREF_1)) |
| *Solanum lycopersicum* | 248 |  | 142 | 21 | 40 | 20 |  | 2 | 1 |  |  |  |  |  |  | ([Yang et al., 2019](#_ENREF_6)) |
| *Oryza sativa* | 281 | 119 |  |  | 23 | 2 |  |  |  |  |  |  |  |  |  | ([Lim et al., 2010](#_ENREF_5)) |
| *Malus domestica* | 367 | 208 |  |  | 35 | 10 | 1 | 11 | 2 | 10 | 44 |  |  |  |  | ([Li et al., 2011](#_ENREF_4)) |
| *Brassica oleracea* | 355 |  | 214 | 36 | 49 | 86 | 11 | 4 | 1 |  |  |  |  |  |  | ([Yang and Lu, 2018](#_ENREF_7)) |
| *Ostreococus tauri* | 25 | 28 |  |  | 7 | 1 |  |  |  |  |  | 1 | 1 | 1 |  | ([Gao et al., 2016](#_ENREF_2)) |

**Reference**

Alam, I., Yang, Y.-Q., Wang, Y., Zhu, M.-L., Wang, H.-B., Chalhoub, B., and Lu, Y.-H. (2017). Genome-wide identification, evolution and expression analysis of RING finger protein genes in Brassica rapa. *Scientific reports* 7**,** 1-11.

Gao, Y., Li, M.-Y., Zhao, J., Zhang, Y.-C., Xie, Q.-J., and Chen, D.-H. (2016). Genome-wide analysis of RING finger proteins in the smallest free-living photosynthetic eukaryote Ostreococus tauri. *Marine genomics* 26**,** 51-61.

Jiménez-López, D., Muñóz-Belman, F., González-Prieto, J.M., Aguilar-Hernández, V., and Guzmán, P. (2018). Repertoire of plant RING E3 ubiquitin ligases revisited: New groups counting gene families and single genes. *PloS one* 13**,** e0203442.

Li, Y., Wu, B., Yu, Y., Yang, G., Wu, C., and Zheng, C. (2011). Genome-wide analysis of the RING finger gene family in apple. *Molecular Genetics and Genomics* 286**,** 81.

Lim, S.D., Yim, W.C., Moon, J.-C., Kim, D.S., Lee, B.-M., and Jang, C.S. (2010). A gene family encoding RING finger proteins in rice: their expansion, expression diversity, and co-expressed genes. *Plant molecular biology* 72**,** 369-380.

Yang, L., Miao, M., Lyu, H., Cao, X., Li, J., Li, Y., Li, Z., and Chang, W. (2019). Genome-Wide Identification, Evolution, and Expression Analysis of RING Finger Gene Family in Solanum lycopersicum. *International Journal of Molecular Sciences* 20**,** 4864.

Yang, Y.-Q., and Lu, Y.-H. (2018). Genome-wide survey, characterization, and expression analysis of RING finger protein genes in Brassica oleracea and their syntenic comparison to Brassica rapa and Arabidopsis thaliana. *Genome* 61**,** 685-697.
