# Supplementary material for: RING Zinc Finger Proteins in Plant Abiotic Stress Tolerance
Source: Front Plant Sci. 2022 Apr 14;13:877011. doi: 10.3389/fpls.2022.877011 (PMC9047180; doi:10.3389/fpls.2022.877011)
Supplement: Supplementary file 2 [file Table_2.DOCX]

**Supplementary Table 2** Amino acid sequence of RING zinc finger proteins expressed on the plasma membrane.

| Proteins | Amino acid sequence | Reference |
| --- | --- | --- |
| AtATL78 | MDEATGETETQDFMNVESFSQL | ([Kim and Kim, 2013](#_ENREF_3);[Suh et al., 2016](#_ENREF_5)) |
| RHA2a | mglqgqlsdvssds | ([Li et al., 2011](#_ENREF_4)) |
| OsRFPv6 | DELREMFGYEEHPY | ([Kim et al., 2021](#_ENREF_2)) |
| OsRDCP1 | rgqvppsmfmsphyvtahnmssrarrhqmevers | ([Bae et al., 2011](#_ENREF_1)) |

**References**

Bae, H., Kim, S.K., Cho, S.K., Kang, B.G., and Kim, W.T. (2011). Overexpression of OsRDCP1, a rice RING domain-containing E3 ubiquitin ligase, increased tolerance to drought stress in rice (Oryza sativa L.). *Plant science* 180**,** 775-782.

Kim, J.H., Lim, S.D., and Jang, C.S. (2021). Oryza sativa, C4HC3-type really interesting new gene (RING), OsRFPv6, is a positive regulator in response to salt stress by regulating Na^+^ absorption. *Physiologia Plantarum*. 173, 883-895.

Kim, S.J., and Kim, W.T. (2013). Suppression of Arabidopsis RING E3 ubiquitin ligase AtATL78 increases tolerance to cold stress and decreases tolerance to drought stress. *FEBS letters* 587**,** 2584-2590.

Li, H., Jiang, H., Bu, Q., Zhao, Q., Sun, J., Xie, Q., and Li, C. (2011). The Arabidopsis RING finger E3 ligase RHA2b acts additively with RHA2a in regulating abscisic acid signaling and drought response. *Plant physiology* 156**,** 550-563.

Suh, J.Y., Kim, S.J., Oh, T.R., Cho, S.K., Yang, S.W., and Kim, W.T. (2016). Arabidopsis Tóxicos en Levadura 78 (AtATL78) mediates ABA-dependent ROS signaling in response to drought stress. *Biochemical and biophysical research communications* 469**,** 8-14.
