# Supplementary material for: RING Zinc Finger Proteins in Plant Abiotic Stress Tolerance
Source: Front Plant Sci. 2022 Apr 14;13:877011. doi: 10.3389/fpls.2022.877011 (PMC9047180; doi:10.3389/fpls.2022.877011)
Supplement: Supplementary file 3 [file Table_3.DOCX]

**Supplementary table 3** Functions of RING zinc finger proteins in abiotic stress responses and mode of actions

| **Gene Name** | **Plant Species** | **Type** | **Function** | **Subcellular Localization** | **References** |
| --- | --- | --- | --- | --- | --- |
| *AtAIRP1* | *A. thaliana* | RING-HC | Positively regulates plant drought stress responses | Cell membrane | ([Ryu et al., 2010](#_ENREF_62)) |
| *AtAIRP2* | *A. thaliana* | RING-HC | Positively regulates plant drought stress responses | Cytoplasm | ([Cho et al., 2011](#_ENREF_8)) |
| *AtAIRP3* | *A. thaliana* | RING-HC | Positively regulates plant drought and salt stress responses | Cell membrane | ([Kim and Kim, 2013a](#_ENREF_24)) |
| *AtAIRP4* | *A. thaliana* | RING-HC | Positively regulates plant drought stress responses | Cytoplasm | ([Yang et al., 2016](#_ENREF_68)) |
| *AtATRF1* | *A. thaliana* | RING-HC | Enhances aluminium tolerance in Arabidopsis | Nucleus | ([Qin et al., 2017](#_ENREF_61)) |
| *AtATL78* | *A. thaliana* | RING-H2 | Positively regulates drought stress but negatively regulates cold stress | Cell membrane | ([Kim and Kim, 2013b](#_ENREF_28);[Suh et al., 2016](#_ENREF_63)) |
| *AtOHRP1* | *A. thaliana* | RING-HC | Positively regulates plant oxidative stress responses |  | ([Li et al., 2013](#_ENREF_34)) |
| *AtPPRT1* | *A. thaliana* | RING-HC | Positively regulates heat stress but negatively regulates drought and salt stress | Mitochondria | ([Pei et al., 2019](#_ENREF_57);[Liu et al., 2020a](#_ENREF_43);[Liu et al., 2020b](#_ENREF_44)) |
| *AtRZF1* | *A. thaliana* | RING-H2 | Positively regulates plant drought stress responses | Nucleus | ([Ju et al., 2013](#_ENREF_20);[Min et al., 2021](#_ENREF_47)) |
| *DRIP1* | *A. thaliana* | RING-HC | Negatively regulates plant drought stress response | Nucleus | ([Qin et al., 2008](#_ENREF_60)) |
| *DRIP2* | *A. thaliana* | RING-HC | Negatively regulates plant drought stress response | Nucleus | ([Qin et al., 2008](#_ENREF_60)) |
| *EMR* | *A. thaliana* | RING-HC | Involved in the degradation of ER-associated protein | Cytoplasm | ([Park et al., 2018a](#_ENREF_51)) |
| *HOS1* | *A. thaliana* | RING-C2 | Decreases plant tolerance to cold stress | Nucleus | ([Ishitani et al., 1998](#_ENREF_17);[Lee et al., 2001](#_ENREF_30);[Dong et al., 2006](#_ENREF_10)) |
| *NCA1* | *A. thaliana* |  | Positively regulates plant oxidative stress responses | Cytoplasm | ([Li et al., 2015](#_ENREF_33)) |
| *NERF* | *A. thaliana* | RING-HC | Regulator of plant tolerance to drought stress | Nucleus | ([Gao et al., 2015](#_ENREF_12)) |
| *RGLG2* | *A. thaliana* | RING-HC | Negatively regulates plant drought stress response | Plasma membrane | ([Cheng et al., 2012](#_ENREF_7);[Yu et al., 2020](#_ENREF_70)) |
| *RHA2a* | *A. thaliana* | RING-H2 | Positively regulates plant drought stress responses and ABA signaling | Plasma membrane and nucleus | ([Li et al., 2011](#_ENREF_32)) |
| *RHA2b* | *A. thaliana* | RING-H2 | Positively regulates plant drought stress responses and ABA signaling | Plasma membrane and nucleus | ([Li et al., 2011](#_ENREF_32)) |
| *Rma1* | *A. thaliana* | RING-HC | Involves in plant drought stress responses by regulates aquaporin levels | Endoplasmic network | ([Matsuda et al., 2001](#_ENREF_46);[Lee et al., 2009](#_ENREF_31)) |
| *SDIR1* | *A. thaliana* | RING-H2 | Positively regulates plant salt and drought stress responses | Plasma membrane | ([Zhang et al., 2007](#_ENREF_77);[Zhang et al., 2015](#_ENREF_74)) |
| *STRF1* | *A. thaliana* | RING-H2 | Monitor intracellular membrane trafficking and ROS production in response to salt stress | Cytoplasm and Plasma membrane | ([Tian et al., 2015](#_ENREF_64)) |
| *XERICO* | *A. thaliana* | RING-H2 | Positively regulates plant drought stress responses |  | ([Ko et al., 2006](#_ENREF_29)) |
| *OsBIRF1* | *O. sativa* | RING-H2 | Enhance plant tolerance to oxidative, drought stress and disease |  | ([Liu et al., 2008](#_ENREF_39)) |
| *OsCOIN* | *O. sativa* | RING-C2 | Positively regulates plant cold,drought and salt stress responses | Nuclear and cytoplasm | ([Liu et al., 2007](#_ENREF_41)) |
| *OsCLR1* | *O. sativa* | RING-H2 | Positively regulates plant salt and drought stress responses | Cytoplasm | ([Park et al., 2019a](#_ENREF_54)) |
| *OsDIS1* | *O. sativa* | RING-H2 | Positively regulates plant drought stress responses | Nucleus | ([Ning et al., 2011a](#_ENREF_48);[Ning et al., 2011b](#_ENREF_49)) |
| *OsDSG1* | *O. sativa* | RING-H2 | Negatively regulates plant drought stress responses. |  | ([Park et al., 2010](#_ENREF_50)) |
| *OsDIRP1* | *O. sativa* | RING-H2 | Plays an opposite role in drought and cold Stress responses as a negative and positive Factor, respectively | Nucleus | ([Cui et al., 2018](#_ENREF_9)) |
| *OsDHSRP1* | *O. sativa* | RING-H2 | Negatively regulates plant drought, heat and salt stress responses. | Microtubule cytoskeleton | ([Kim et al., 2020](#_ENREF_26)) |
| *OsHCI1* | *O. sativa* | RING-HC | Positively regulates plant heat stress responses | Golgi apparatus | ([Lim et al., 2013](#_ENREF_37)) |
| *OsHIRP1* | *O. sativa* | RING-HC | Positively regulates plant drought stress responses | Nuclear and cytoplasm | ([Kim et al., 2019](#_ENREF_25)) |
| *OsHTAS* | *O. sativa* | RING-H2 | Positively regulates plant heat stress responses | Nuclear and cytoplasm | ([Liu et al., 2016](#_ENREF_40)) |
| *OsMAR1* | *O. sativa* | RING-H2 | Negatively regulates plant salt stress responses. | Related to the microtubules | ([Park et al., 2018b](#_ENREF_52)) |
| *OsRDCP1* | *O. sativa* | RING-HC | Increased tolerance to drought stress in rice | Plasma membrane | ([Bae et al., 2011](#_ENREF_4)) |
| *OsRHP1* | *O. sativa* | RING-H2 | Positively regulates plant drought and salt stress responses |  | ([Zeng et al., 2014](#_ENREF_72)) |
| *OsRZFP34* | *O. sativa* | RING-HC | Positively regulates plant heat stress responses |  | ([Hsu et al., 2014](#_ENREF_15)) |
| *OsRFPv6* | *O. sativa* | RING-HC | Positively regulates plant salt stress responses | Cytoplasm and Plasma membrane | ([Kim et al., 2021](#_ENREF_27)) |
| *OsSADR1* | *O. sativa* | RING-H2 | Negatively regulates plant salt stress responses | Nucleus | ([Park et al., 2018c](#_ENREF_53)) |
| *OsSIRH2-14* | *O. sativa* | RING-H2 | Positively regulates plant salt stress responses | Plasma membrane, cytoplasm, Golgi | ([Park et al., 2019b](#_ENREF_55)) |
| *OsSIRP1* | *O. sativa* | RING-HC | Negatively regulates plant salt stress responses | Cytoplasm | ([Hwang et al., 2016](#_ENREF_16)) |
| *OsSIRP2* | *O. sativa* | RING-HC | Positively regulates plant salt stress responses | Nucleus | ([Chapagain et al., 2018](#_ENREF_6)) |
| *OsSIRP3* | *O. sativa* | RING-H2 | Negatively regulates plant salt stress responses | Cytoplasm | ([Park et al., 2018d](#_ENREF_56)) |
| *OsSIRP4* | *O. sativa* | RING-HC | Negatively regulates plant salt stress responses | Cytoplasm and Plasma membrane | ([Kim and Jang, 2021](#_ENREF_23)) |
| *CaAIRF1* | *C. annuum L.* | RING-HC | Positively regulates plant drought stress responses and ABA signaling | Nucleus | ([Lim et al., 2017](#_ENREF_35)) |
| *CaASRF1* | *C. annuum L.* | RING-H2 | Positively regulates plant drought stress responses | Nuclear and cytoplasm | ([Joo et al., 2019](#_ENREF_18)) |
| *CaATIR1* | *C. annuum L.* | RING-H2 | Positively regulates plant drought stress responses and ABA signaling | Nucleus | ([Joo et al., 2020](#_ENREF_19)) |
| *CaDSR1* | *C. annuum L.* | RING-H2 | Negatively regulates plant salt stress responses | Nuclear and cytoplasm | ([Lim et al., 2018](#_ENREF_36)) |
| *CaRFP1* | *C. annuum L.* | RING-HC | Positively regulates plant osmotic stress responses and enhances disease susceptibility |  | ([Hong et al., 2007](#_ENREF_14)) |
| *CaRZFP1* | *C. annuum L.* | RING-HC | Regulator of root development |  | ([Zeba et al., 2006](#_ENREF_71)) |
| *Rma1H1* | *C. annuum L.* | RING-HC | Positively regulates plant drought stress responses | Endoplasmic network | ([Lee et al., 2009](#_ENREF_31)) |
| *ZmRFP1* | *Z. mays L.* | RING-H2 | Regulator of plant tolerance to drought stress | Plasma membrane | ([Xia et al., 2012](#_ENREF_66)) |
| *ZmXerico1* | *Z. mays L.* | RING-H2 | Positively regulates plant drought stress responses | Cytoplasm | ([Gao et al., 2012](#_ENREF_13);[Brugière et al., 2017](#_ENREF_5)) |
| *ZmXerico2* | *Z. mays L.* | RING-H2 | Positively regulates plant drought stress responses | Cytoplasm | ([Brugière et al., 2017](#_ENREF_5)) |
| *TaDIS1* | *T. aestivuml* | RING-HC | Negatively regulates plant drought stress responses | Golgi apparatus | ([Liu et al., 2018](#_ENREF_42);[Lv et al., 2020](#_ENREF_45)) |
| *TaZnF* | *T. aestivuml* | RING-HC | Positively regulates plant various stress responses |  | ([Agarwal and Khurana, 2018](#_ENREF_1);[2020](#_ENREF_2)) |
| *TaRZF70* | *T. aestivuml* | RING-H2 | Involved in plant drought stress |  | ([Kam et al., 2007](#_ENREF_22)) |
| *AdZFP1* | *A.dracunculus L.* | RING-HC | Positively regulates plant drought stress responses |  | ([Yang et al., 2008](#_ENREF_69)) |
| *BrRZFP1* | *B. rapa* | RING-HC | Positively regulates plant salt and drought stress responses | Cytoplasm and Plasma membrane | ([Jung et al., 2013](#_ENREF_21)) |
| *BdRHP1* | *B.distachyon* | RING-H2 | Positively regulates plant drought stress responses |  | ([ZENG et al., 2019](#_ENREF_73)) |
| *GmARI1* | *G. max* | RING-HC | Enhances plant tolerance to aluminum stress | Nucleus | ([Zhang et al., 2014](#_ENREF_76)) |
| *GmRZFP1* | *G. max* | RING-HC | Positively regulates plant oxidative stress responses | Nucleus | ([WU et al., 2010](#_ENREF_65)) |
| *MeRZF* | *M. esculenta* | RING-H2 | Involved in plant salt stress | Plasma membrane | ([Dos Reis et al., 2012](#_ENREF_11)) |
| *MfSTMIR* | *M. falcata* | RING-H2 | Eliminate endoplasmic reticulum misfolded proteins to alleviate salt stress damage to plants | Endoplasmic network | ([Zhang et al., 2019](#_ENREF_75)) |
| *NtRHF1* | *N. tabacum* | RING-H2 | Positively regulates plant drought stress responses |  | ([Xia et al., 2013](#_ENREF_67)) |
| *SpRing* | *S.lycopersicum* | RING-H2 | Positively regulates plant salt stress responses | Endoplasmic network | ([Qi et al., 2016](#_ENREF_58)) |
| *SlRING1* | *S.lycopersicum L.* |  | Positively regulates plant cadmium (Cd) tolerance | Plasma membrane and nucleus | ([Ahammed et al., 2020](#_ENREF_3)) |
| *StRFP2* | *S.tuberosum L.* | RING-H2 | Positively regulates plant drought stress responses | Cytoplasm and Plasma membrane | ([Qi et al., 2020](#_ENREF_59)) |
| *SbHCI1* | *S. bicolor* | RING-HC | Positively regulates plant heat stress responses | Cytoplasm and golgi apparatus | ([Lim et al., 2020](#_ENREF_38)) |

**References**

Agarwal, P., and Khurana, P. (2018). Characterization of a novel zinc finger transcription factor (TaZnF) from wheat conferring heat stress tolerance in Arabidopsis. *Cell Stress and Chaperones* 23**,** 253-267.

Agarwal, P., and Khurana, P. (2020). TaZnF, a C3HC4 type RING zinc finger protein from Triticum aestivum is involved in dehydration and salinity stress. *Journal of Plant Biochemistry and Biotechnology* 29**,** 395-406.

Ahammed, G.J., Li, C.X., Li, X., Liu, A., Chen, S., and Zhou, J. (2020). Overexpression of tomato RING E3 ubiquitin ligase gene SlRING1 confers cadmium tolerance by attenuating cadmium accumulation and oxidative stress. *Physiologia Plantarum* 173**,** 449-459.

Bae, H., Kim, S.K., Cho, S.K., Kang, B.G., and Kim, W.T. (2011). Overexpression of OsRDCP1, a rice RING domain-containing E3 ubiquitin ligase, increased tolerance to drought stress in rice (Oryza sativa L.). *Plant science* 180**,** 775-782.

Brugière, N., Zhang, W., Xu, Q., Scolaro, E.J., Lu, C., Kahsay, R.Y., Kise, R., Trecker, L., Williams, R.W., and Hakimi, S. (2017). Overexpression of RING domain E3 ligase ZmXerico1 confers drought tolerance through regulation of ABA homeostasis. *Plant physiology* 175**,** 1350-1369.

Chapagain, S., Park, Y.C., Kim, J.H., and Jang, C.S. (2018). Oryza sativa salt-induced RING E3 ligase 2 (OsSIRP2) acts as a positive regulator of transketolase in plant response to salinity and osmotic stress. *Planta* 247**,** 925-939.

Cheng, M.-C., Hsieh, E.-J., Chen, J.-H., Chen, H.-Y., and Lin, T.-P. (2012). Arabidopsis RGLG2, functioning as a RING E3 ligase, interacts with AtERF53 and negatively regulates the plant drought stress response. *Plant physiology* 158**,** 363-375.

Cho, S.K., Ryu, M.Y., Seo, D.H., Kang, B.G., and Kim, W.T. (2011). The Arabidopsis RING E3 ubiquitin ligase AtAIRP2 plays combinatory roles with AtAIRP1 in abscisic acid-mediated drought stress responses. *Plant physiology* 157**,** 2240-2257.

Cui, L.H., Min, H.J., Byun, M.Y., Oh, H.G., and Kim, W.T. (2018). OsDIRP1, a putative RING E3 ligase, plays an opposite role in drought and cold stress responses as a negative and positive factor, respectively, in rice (Oryza sativa L.). *Frontiers in plant science* 9**,** 1797.

Dong, C.-H., Agarwal, M., Zhang, Y., Xie, Q., and Zhu, J.-K. (2006). The negative regulator of plant cold responses, HOS1, is a RING E3 ligase that mediates the ubiquitination and degradation of ICE1. *Proceedings of the National Academy of Sciences* 103**,** 8281-8286.

Dos Reis, S.P., Tavares, L.D.S.C., Costa, C.D.N.M., Santa Brígida, A.B., and De Souza, C.R.B. (2012). Molecular cloning and characterization of a novel RING zinc-finger protein gene up-regulated under in vitro salt stress in cassava. *Molecular biology reports* 39**,** 6513-6519.

Gao, W., Liu, W., Zhao, M., and Li, W.-X. (2015). NERF encodes a RING E3 ligase important for drought resistance and enhances the expression of its antisense gene NFYA5 in Arabidopsis. *Nucleic acids research* 43**,** 607-617.

Gao, Y., Li, H., Deng, D., Chen, S., Jiang, W., and Chen, J. (2012). Characterization and expression analysis of the maize RING-H2 finger protein gene ZmXERICO responsive to plant hormones and abiotic stresses. *Acta Physiologiae Plantarum* 34**,** 1529-1535.

Hong, J.K., Choi, H.W., Hwang, I.S., and Hwang, B.K. (2007). Role of a novel pathogen-induced pepper C3–H–C4 type RING-finger protein gene, CaRFP1, in disease susceptibility and osmotic stress tolerance. *Plant molecular biology* 63**,** 571-588.

Hsu, K.-H., Liu, C.-C., Wu, S.-J., Kuo, Y.-Y., Lu, C.-A., Wu, C.-R., Lian, P.-J., Hong, C.-Y., Ke, Y.-T., and Huang, J.-H. (2014). Expression of a gene encoding a rice RING zinc-finger protein, OsRZFP34, enhances stomata opening. *Plant molecular biology* 86**,** 125-137.

Hwang, S.G., Kim, J.J., Lim, S.D., Park, Y.C., Moon, J.C., and Jang, C.S. (2016). Molecular dissection of Oryza sativa salt‐induced RING Finger Protein 1 (OsSIRP1): possible involvement in the sensitivity response to salinity stress. *Physiologia plantarum* 158**,** 168-179.

Ishitani, M., Xiong, L., Lee, H., Stevenson, B., and Zhu, J.-K. (1998). HOS1, a genetic locus involved in cold-responsive gene expression in Arabidopsis. *The Plant Cell* 10**,** 1151-1161.

Joo, H., Lim, C.W., and Lee, S.C. (2019). A pepper RING‐type E3 ligase, CaASRF1, plays a positive role in drought tolerance via modulation of CaAIBZ1 stability. *The Plant Journal* 98**,** 5-18.

Joo, H., Lim, C.W., and Lee, S.C. (2020). The pepper RING‐type E3 ligase, CaATIR1, positively regulates abscisic acid signalling and drought response by modulating the stability of CaATBZ1. *Plant, Cell & Environment* 43**,** 1911-1924.

Ju, H.-W., Min, J.-H., Chung, M.-S., and Kim, C.S. (2013). The atrzf1 mutation of the novel RING-type E3 ubiquitin ligase increases proline contents and enhances drought tolerance in Arabidopsis. *Plant Science* 203**,** 1-7.

Jung, Y.-J., Lee, I.-H., Nou, I.-S., Lee, K.-D., Rashotte, A.M., and Kang, K.-K. (2013). BrRZFP1 a Brassica rapa C3HC4‐type RING zinc finger protein involved in cold, salt and dehydration stress. *Plant Biology* 15**,** 274-283.

Kam, J., Gresshoff, P., Shorter, R., and Xue, G.-P. (2007). Expression analysis of RING zinc finger genes from Triticum aestivum and identification of TaRZF70 that contains four RING-H2 domains and differentially responds to water deficit between leaf and root. *Plant science* 173**,** 650-659.

Kim, J.H., and Jang, C.S. (2021). E3 ligase, the Oryza sativa salt-induced RING finger protein 4 (OsSIRP4), negatively regulates salt stress responses via degradation of the OsPEX11-1 protein. *Plant Molecular Biology* 105**,** 231-245.

Kim, J.H., and Kim, W.T. (2013a). The Arabidopsis RING E3 ubiquitin ligase AtAIRP3/LOG2 participates in positive regulation of high-salt and drought stress responses. *Plant physiology* 162**,** 1733-1749.

Kim, J.H., Lim, S.D., and Jang, C.S. (2019). Oryza sativa heat-induced RING finger protein 1 (OsHIRP1) positively regulates plant response to heat stress. *Plant molecular biology* 99**,** 545-559.

Kim, J.H., Lim, S.D., and Jang, C.S. (2020). Oryza sativa drought-, heat-, and salt-induced RING finger protein 1 (OsDHSRP1) negatively regulates abiotic stress-responsive gene expression. *Plant Molecular Biology***,** 1-18.

Kim, J.H., Lim, S.D., and Jang, C.S. (2021). Oryza sativa, C4HC3‐type really interesting new gene (RING), OsRFPv6, is a positive regulator in response to salt stress by regulating Na^+^ absorption. *Physiologia Plantarum* 173**,** 883-895

Kim, S.J., and Kim, W.T. (2013b). Suppression of Arabidopsis RING E3 ubiquitin ligase AtATL78 increases tolerance to cold stress and decreases tolerance to drought stress. *FEBS letters* 587**,** 2584-2590.

Ko, J.H., Yang, S.H., and Han, K.H. (2006). Upregulation of an Arabidopsis RING‐H2 gene, XERICO, confers drought tolerance through increased abscisic acid biosynthesis. *The Plant Journal* 47**,** 343-355.

Lee, H., Xiong, L., Gong, Z., Ishitani, M., Stevenson, B., and Zhu, J.-K. (2001). The Arabidopsis HOS1 gene negatively regulates cold signal transduction and encodes a RING finger protein that displays cold-regulated nucleo–cytoplasmic partitioning. *Genes & development* 15**,** 912-924.

Lee, H.K., Cho, S.K., Son, O., Xu, Z., Hwang, I., and Kim, W.T. (2009). Drought stress-induced Rma1H1, a RING membrane-anchor E3 ubiquitin ligase homolog, regulates aquaporin levels via ubiquitination in transgenic Arabidopsis plants. *The Plant Cell* 21**,** 622-641.

Li, H., Jiang, H., Bu, Q., Zhao, Q., Sun, J., Xie, Q., and Li, C. (2011). The Arabidopsis RING finger E3 ligase RHA2b acts additively with RHA2a in regulating abscisic acid signaling and drought response. *Plant physiology* 156**,** 550-563.

Li, J., Liu, J., Wang, G., Cha, J.-Y., Li, G., Chen, S., Li, Z., Guo, J., Zhang, C., and Yang, Y. (2015). A chaperone function of NO CATALASE ACTIVITY1 is required to maintain catalase activity and for multiple stress responses in Arabidopsis. *The Plant Cell* 27**,** 908-925.

Li, J., Yang, F., Liu, Z., Yang, Y., and Li, X. (2013). First exploration on function of Arabidopsis AtOHRP1 in response to oxidative stress. *Journal of Agricultural Science and Technology (Beijing)* 15**,** 93-99.

Lim, C.W., Baek, W., and Lee, S.C. (2017). The pepper RING-type E3 ligase CaAIRF1 regulates ABA and drought signaling via CaADIP1 protein phosphatase degradation. *Plant physiology* 173**,** 2323-2339.

Lim, C.W., Baek, W., and Lee, S.C. (2018). Roles of pepper bZIP protein Ca DILZ 1 and its interacting partner RING‐type E3 ligase Ca DSR 1 in modulation of drought tolerance. *The Plant Journal* 96**,** 452-467.

Lim, S.D., Cho, H.Y., Park, Y.C., Ham, D.J., Lee, J.K., and Jang, C.S. (2013). The rice RING finger E3 ligase, OsHCI1, drives nuclear export of multiple substrate proteins and its heterogeneous overexpression enhances acquired thermotolerance. *Journal of experimental botany* 64**,** 2899-2914.

Lim, S.D., Oh, D.G., Park, Y.C., and Jang, C.S. (2020). Molecular characterization of a RING E3 ligase SbHCI1 in sorghum under heat and abscisic acid stress. *Planta* 252**,** 1-18.

Liu, H., Zhang, H., Yang, Y., Li, G., Yang, Y., Basnayake, B.V.S., Li, D., and Song, F. (2008). Functional analysis reveals pleiotropic effects of rice RING-H2 finger protein gene OsBIRF1 on regulation of growth and defense responses against abiotic and biotic stresses. *Plant molecular biology* 68**,** 17-30.

Liu, J., Zhang, C., Wei, C., Liu, X., Wang, M., Yu, F., Xie, Q., and Tu, J. (2016). The RING finger ubiquitin E3 ligase OsHTAS enhances heat tolerance by promoting H2O2-induced stomatal closure in rice. *Plant Physiology* 170**,** 429-443.

Liu, K., Wang, L., Xu, Y., Chen, N., Ma, Q., Li, F., and Chong, K. (2007). Overexpression of OsCOIN, a putative cold inducible zinc finger protein, increased tolerance to chilling, salt and drought, and enhanced proline level in rice. *Planta* 226**,** 1007-1016.

Liu, Y., Li, L., Zhang, L., Lv, Q., Zhao, Y., and Li, X. (2018). Isolation and identification of wheat gene TaDIS1 encoding a RING finger domain protein, which negatively regulates drought stress tolerance in transgenic Arabidopsis. *Plant Science* 275**,** 49-59.

Liu, Y., Pei, L., Xiao, S., Peng, L., Liu, Z., Li, X., Yang, Y., and Wang, J. (2020a). AtPPRT1 negatively regulates salt stress response in Arabidopsis seedlings. *Plant signaling & behavior* 15**,** 1732103.

Liu, Y., Xiao, S., Sun, H., Pei, L., Liu, Y., Peng, L., Gao, X., and Wang, J. (2020b). AtPPRT1, an E3 ubiquitin ligase, enhances the thermotolerance in arabidopsis. *Plants* 9**,** 1074.

Lv, Q., Zhang, L., Zan, T., Li, L., and Li, X. (2020). Wheat RING E3 ubiquitin ligase TaDIS1 degrade TaSTP via the 26S proteasome pathway. *Plant Science* 296**,** 110494.

Matsuda, N., Suzuki, T., Tanaka, K., and Nakano, A. (2001). Rma1, a novel type of RING finger protein conserved from Arabidopsis to human, is a membrane-bound ubiquitin ligase. *Journal of cell science* 114**,** 1949-1957.

Min, J.-H., Park, C.-R., Chung, J.-S., and Kim, C.S. (2021). Arabidopsis thaliana Ubiquitin-Associated Protein 1 (AtUAP1) Interacts with Arabidopsis thaliana Ring Zinc Finger 1 (AtRZF1) to Negatively Regulate Dehydration Response. *Plant and Cell Physiology* 62**,**1044-1057.

Ning, Y., Jantasuriyarat, C., Zhao, Q., Zhang, H., Chen, S., Liu, J., Liu, L., Tang, S., Park, C.H., and Wang, X. (2011a). The SINA E3 ligase OsDIS1 negatively regulates drought response in rice. *Plant Physiology* 157**,** 242-255.

Ning, Y., Xie, Q., and Wang, G.-L. (2011b). OsDIS1-mediated stress response pathway in rice. *Plant signaling & behavior* 6**,** 1684-1686.

Park, G.-G., Park, J.-J., Yoon, J., Yu, S.-N., and An, G. (2010). A RING finger E3 ligase gene, Oryza sativa Delayed Seed Germination 1 (OsDSG1), controls seed germination and stress responses in rice. *Plant molecular biology* 74**,** 467-478.

Park, J.H., Kang, C.H., Nawkar, G.M., Lee, E.S., Paeng, S.K., Chae, H.B., Chi, Y.H., Kim, W.Y., Yun, D.J., and Lee, S.Y. (2018a). EMR, a cytosolic‐abundant ring finger E3 ligase, mediates ER‐associated protein degradation in Arabidopsis. *New Phytologist* 220**,** 163-177.

Park, Y.C., Chapagain, S., and Jang, C.S. (2018b). The microtubule-associated RING finger protein 1 (OsMAR1) acts as a negative regulator for salt-stress response through the regulation of OCPI2 (O. sativa chymotrypsin protease inhibitor 2). *Planta* 247**,** 875-886.

Park, Y.C., Chapagain, S., and Jang, C.S. (2018c). A negative regulator in response to salinity in rice: Oryza sativa Salt-, ABA-and drought-induced RING Finger Protein 1 (OsSADR1). *Plant and Cell Physiology* 59**,** 575-589.

Park, Y.C., Choi, S.Y., Kim, J.H., and Jang, C.S. (2019a). Molecular functions of rice cytosol-localized RING finger protein 1 in response to salt and drought and comparative analysis of its grass orthologs. *Plant and Cell Physiology* 60**,** 2394-2409.

Park, Y.C., Lim, S.D., Moon, J.C., and Jang, C.S. (2019b). A rice really interesting new gene H 2‐type E 3 ligase, OsSIRH2‐14, enhances salinity tolerance via ubiquitin/26 S proteasome‐mediated degradation of salt‐related proteins. *Plant, cell & environment* 42**,** 3061-3076.

Park, Y.C., Moon, J.-C., Chapagain, S., Oh, D.G., Kim, J.J., and Jang, C.S. (2018d). Role of salt-induced RING finger protein 3 (OsSIRP3), a negative regulator of salinity stress response by modulating the level of its target proteins. *Environmental and experimental botany* 155**,** 21-30.

Pei, L., Peng, L., Wan, X., Xiong, J., Liu, Z., Li, X., Yang, Y., and Wang, J. (2019). Expression pattern and function analysis of AtPPRT1, a novel negative regulator in ABA and drought stress responses in Arabidopsis. *International journal of molecular sciences* 20**,** 394.

Qi, S., Lin, Q., Zhu, H., Gao, F., Zhang, W., and Hua, X. (2016). The RING finger E3 ligase SpRing is a positive regulator of salt stress signaling in salt-tolerant wild tomato species. *Plant and Cell Physiology* 57**,** 528-539.

Qi, X., Tang, X., Liu, W., Fu, X., Luo, H., Ghimire, S., Zhang, N., and Si, H. (2020). A potato RING-finger protein gene StRFP2 is involved in drought tolerance. *Plant Physiology and Biochemistry* 146**,** 438-446.

Qin, F., Sakuma, Y., Tran, L.-S.P., Maruyama, K., Kidokoro, S., Fujita, Y., Fujita, M., Umezawa, T., Sawano, Y., and Miyazono, K.-I. (2008). Arabidopsis DREB2A-interacting proteins function as RING E3 ligases and negatively regulate plant drought stress–responsive gene expression. *The plant cell* 20**,** 1693-1707.

Qin, X., Huang, S., Liu, Y., Bian, M., Shi, W., Zuo, Z., and Yang, Z. (2017). Overexpression of A RING finger ubiquitin ligase gene AtATRF1 enhances aluminium tolerance in Arabidopsis thaliana. *Journal of Plant Biology* 60**,** 66-74.

Ryu, M.Y., Cho, S.K., and Kim, W.T. (2010). The Arabidopsis C3H2C3-type RING E3 ubiquitin ligase AtAIRP1 is a positive regulator of an abscisic acid-dependent response to drought stress. *Plant physiology* 154**,** 1983-1997.

Suh, J.Y., Kim, S.J., Oh, T.R., Cho, S.K., Yang, S.W., and Kim, W.T. (2016). Arabidopsis Tóxicos en Levadura 78 (AtATL78) mediates ABA-dependent ROS signaling in response to drought stress. *Biochemical and biophysical research communications* 469**,** 8-14.

Tian, M., Lou, L., Liu, L., Yu, F., Zhao, Q., Zhang, H., Wu, Y., Tang, S., Xia, R., and Zhu, B. (2015). The RING finger E3 ligase STRF1 is involved in membrane trafficking and modulates salt‐stress response in Arabidopsis thaliana. *The Plant Journal* 82**,** 81-92.

Wu, X.-C., Cao, X.-Y., Chen, M., Zhang, X.-K., Liu, Y.-N., Xu, Z.-S., Li, L.-C., and Ma, Y.-Z. (2010). Isolation and Expression Pattern Assay of a C3HC4-type RING Zinc Finger Protein Gene GmRZFP1 in Glycine max (L.). *Journal of Plant Genetic Resources* 11**,** 343-348.

Xia, Z., Liu, Q., Wu, J., and Ding, J. (2012). ZmRFP1, the putative ortholog of SDIR1, encodes a RING-H2 E3 ubiquitin ligase and responds to drought stress in an ABA-dependent manner in maize. *Gene* 495**,** 146-153.

Xia, Z., Su, X., Liu, J., and Wang, M. (2013). The RING-H2 finger gene 1 (RHF1) encodes an E3 ubiquitin ligase and participates in drought stress response in Nicotiana tabacum. *Genetica* 141**,** 11-21.

Yang, L., Liu, Q., Liu, Z., Yang, H., Wang, J., Li, X., and Yang, Y. (2016). Arabidopsis C3HC4‐RING finger E3 ubiquitin ligase AtAIRP4 positively regulates stress‐responsive abscisic acid signaling. *Journal of integrative plant biology* 58**,** 67-80.

Yang, X., Sun, C., Hu, Y., and Lin, Z. (2008). Molecular cloning and characterization of a gene encoding RING zinc finger ankyrin protein from drought-tolerant Artemisia desertorum. *Journal of biosciences* 33**,** 103-112.

Yu, J., Kang, L., Li, Y., Wu, C., Zheng, C., Liu, P., and Huang, J. (2020). RING finger protein RGLG1 and RGLG2 negatively modulate MAPKKK18 mediated drought stress tolerance in Arabidopsis. *Journal of Integrative Plant Biology* 63**,** 484-493.

Zeba, N., Ashrafuzzaman, M., and Hong, C.B. (2006). Molecular characterization of theCapsicum annuum RING zinc finger protein 1 (CaRZFP1) gene induced by abiotic stresses. *Journal of Plant Biology* 49**,** 484-490.

Zeng, D.-E., Hou, P., Xiao, F., and Liu, Y. (2014). Overexpressing a novel RING-H2 finger protein gene, OsRHP1, enhances drought and salt tolerance in rice (Oryza sativa L.). *Journal of Plant Biology* 57**,** 357-365.

Zeng, D., Cai, Z., and Liu, Y. (2019). Overexpression of BdRHP1 improves drought tolerance and reduces yield loss in rice. *Biologia plantarum* 63**,** 371-379.

Zhang, H., Cui, F., Wu, Y., Lou, L., Liu, L., Tian, M., Ning, Y., Shu, K., Tang, S., and Xie, Q. (2015). The RING finger ubiquitin E3 ligase SDIR1 targets SDIR1-INTERACTING PROTEIN1 for degradation to modulate the salt stress response and ABA signaling in Arabidopsis. *The Plant Cell* 27**,** 214-227.

Zhang, R., Chen, H., Duan, M., Zhu, F., Wen, J., Dong, J., and Wang, T. (2019). Medicago falcata MfSTMIR, an E3 ligase of endoplasmic reticulum‐associated degradation, is involved in salt stress response. *The Plant Journal* 98**,** 680-696.

Zhang, X., Wang, N., Chen, P., Gao, M., Liu, J., Wang, Y., Zhao, T., Li, Y., and Gai, J. (2014). Overexpression of a soybean ariadne-like ubiquitin ligase gene GmARI1 enhances aluminum tolerance in Arabidopsis. *PLoS One* 9**,** e111120.

Zhang, Y., Yang, C., Li, Y., Zheng, N., Chen, H., Zhao, Q., Gao, T., Guo, H., and Xie, Q. (2007). SDIR1 is a RING finger E3 ligase that positively regulates stress-responsive abscisic acid signaling in Arabidopsis. *The Plant Cell* 19**,** 1912-1929.
